# Correlation of Norovirus in Wastewater with Gastroenteritis-Related Hospitalizations, New York, USA, 2022–2024 

**DOI:** 10.3201/eid3213.260472

**Published:** 2026-08

**Authors:** Ozioma F. Nwabor, Patrick Bryant, Dustin Hill, Mohammed Alazawi, Lynsey Schoultz, Aishwarya Jadhav, Ian Bradley, Mila Neyra-Blatz, Yinyin Ye, Kirsten St. George, David A. Larsen

**Affiliations:** Syracuse University, Syracuse, New York, USA (O.F. Nwabor, D. Hill, M. Neyra-Blatz, D.A. Larsen); New York State Department of Health, Albany, New York, USA (P. Bryant, M. Alazawi, L. Schoultz, A. Jadhav, K. St. George); University at Buffalo, Buffalo, New York, USA (I. Bradley, Y. Ye); New York State Department of Health, Slingerlands, New York, USA (K. St. George); University at Albany, Albany (K. St. George)

**Keywords:** norovirus, viruses, wastewater surveillance, enteric infections, wastewater-based epidemiology, surveillance, acute gastroenteritis, hospitalization, New York, United States

## Abstract

Norovirus is a substantial public health burden, yet surveillance is challenging because infections are underreported. Wastewater surveillance offers a complementary approach. We quantified norovirus genogroups GI and GII from 2,823 wastewater samples collected in New York, USA, during September 2022–July 2024. Across 4 counties in New York, we detected norovirus GI in 100% of samples and GII in 92%–100%. Both genogroups exhibited strong seasonal trends. During the study period, 499 norovirus-specific and 50,004 all-cause acute gastroenteritis hospitalizations occurred. Weekly correlations between GII concentrations and norovirus-specific hospitalizations showed moderate positive relationships (ρ = 0.23–0.28); GI concentrations showed weak correlations (ρ = −0.18 to 0.06). Correlations between norovirus in wastewater and all-cause acute gastroenteritis hospitalizations were weaker. Although wastewater surveillance reliably detects seasonal norovirus circulation in the population, correlations with hospitalizations were moderate. GII showed stronger associations with clinical outcomes than GI, supporting the importance of genogroup-specific monitoring.

Noroviruses are genetically diverse icosahedral viruses belonging to the family Caliciviridae; they consist of a single-stranded, positive-sense RNA genome. They are a leading cause of diarrheal disease. and can infect a wide variety of hosts (*1*). Norovirus consists of 10 genogroups (GI–GX) and 49 genotypes. Genogroups GI, GII, GIV, GVIII and GIX are known to infect humans, whereas genogroup GIII primarily infects cows and GV mice (*1*–*4*). Norovirus infections are highly contagious, causing gastroenteritis that is typically mild and self-limiting in healthy adults but can be severe and even fatal in children, elderly, and immunocompromised persons. In the United States, outbreaks peak during November–April (*5*,*6*) and cause $2.3 billion in direct medical costs and $1.4–$20.7 billion in loss of productivity annually (*7*).

As of August 2026, norovirus surveillance relies primarily on either outbreak detection with case investigations or individual case reporting. Most norovirus infections in healthy adults are either asymptomatic or self-limiting and thus not tested, leading to a large burden of unreported incidence (*1*,*8*,*9*). In most US jurisdictions norovirus is not a notifiable disease. Poor clinical surveillance provides inaccurate estimates of norovirus incidence, which leads to lower prioritization for prevention and control. Understanding the true epidemiologic burden of norovirus requires an approach that addresses those limitations. Wastewater surveillance is one potential data source; it does not depend on symptomatic persons seeking medical care but enables community-level infectious disease surveillance (*10*).

The level of norovirus RNA in wastewater has been associated with the monthly incidence of acute gastroenteritis hospitalizations across diverse geographic regions (*11*). Several studies have found a strong correlation between norovirus genogroup–specific concentrations in wastewater and clinical outcomes (*12**–**15*). GII strains are more strongly associated with large-scale outbreaks and severe gastroenteritis (*16*), but they infect animals as well as humans, which could decrease the utility of wastewater surveillance (*17*); GI strains are often linked to sporadic cases and specifically infect humans (*18*). Here, we investigate epidemiologic trends of norovirus GI and GII by measuring the concentration of both genogroups in wastewater across 4 counties in the state of New York, USA. We hypothesized that higher wastewater concentrations would correlate with an increase in norovirus and acute gastroenteritis hospitalizations, especially during colder months, when norovirus activity tends to be higher, in light of previous studies linking wastewater viral concentrations to disease incidence. We correlated genogroup-specific norovirus wastewater concentrations with hospitalized cases of acute gastroenteritis to better understand the potential utility of wastewater surveillance to support norovirus surveillance.

## Methods

### Study Design

**Figure 1:**
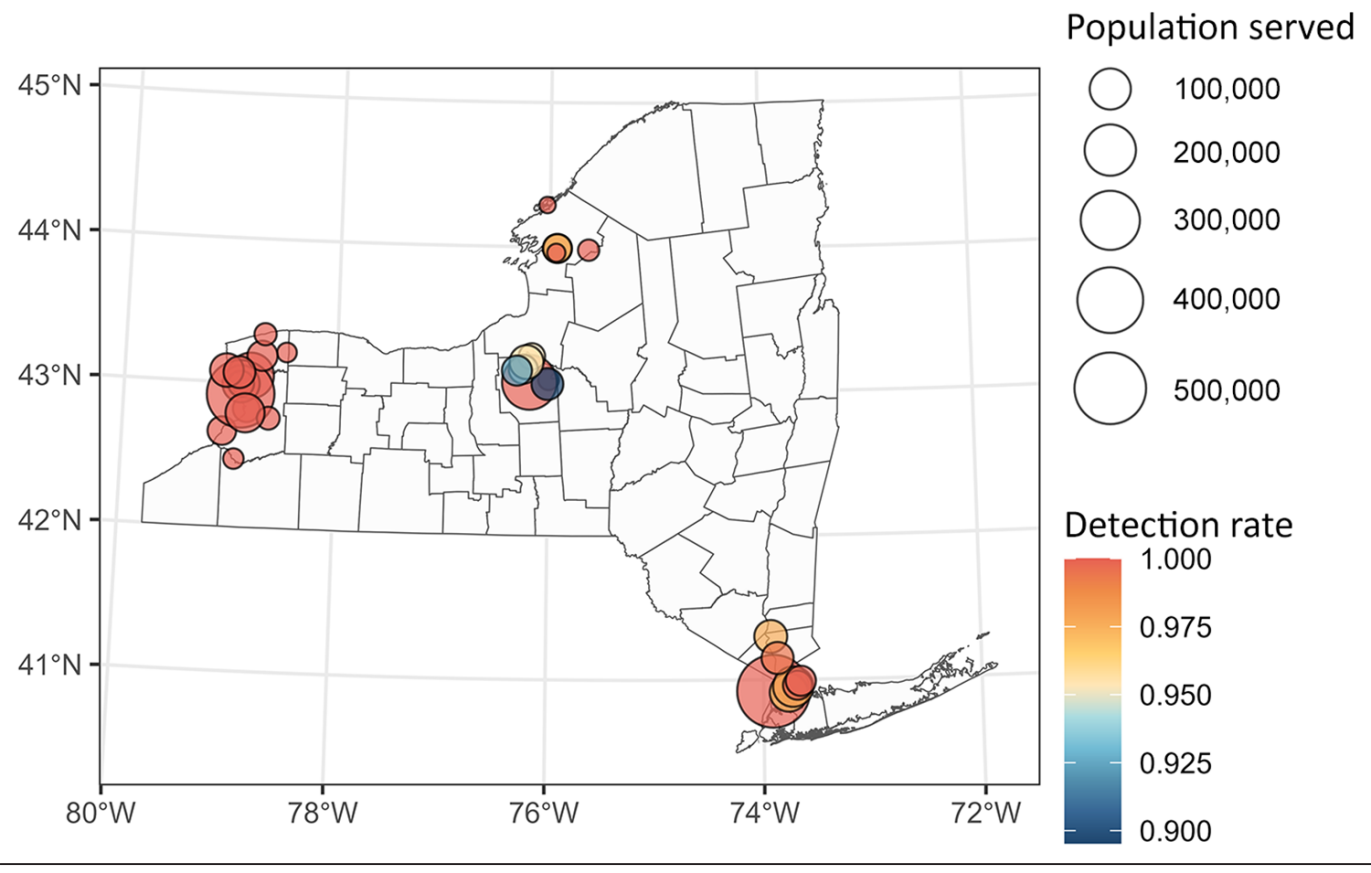
Spatial distribution of sampled wastewater treatment plants in study of correlation between norovirus in wastewater with norovirus-attributable and general acute gastroenteritis–related hospitalizations, New York, USA, 2022–2024. Norovirus genotype I was detected in every sample across the study. Colors indicate detection rate (proportion of samples tested that are positive) of norovirus genotype II across the wastewater treatment plants included in this study.

We designed an ecologic study to assess the relationship between the concentration of norovirus in wastewater and the incidence of norovirus-attributable and all-cause acute gastroenteritis-related hospitalizations. We conducted the study during September 2022–July 2024, taking samples from 24 wastewater treatment plants in 4 counties in New York: Erie (Buffalo, NY, USA), Onondaga (Syracuse, NY, USA), Jefferson (Watertown, NY, USA), and Westchester (north of New York, NY, USA) (Figure 1).

### Setting

The New York State Wastewater Surveillance Network was initiated in March 2020 as a tool to track and address emerging diseases and monitor evolving public health threats (*19*); its purpose is to provide population-level data on community health status and disease burden through sampling and analysis of wastewater from across the state. As part of the study, we sampled 9 treatment plants in Erie County; for each, capacity was 2.20–180 million gallons permitted discharge per day (MGD) and population served was 2,854–431,141, covering ≈83.4% of the county population. In Westchester County, we sampled 7 treatment plants, each with a capacity of 5.0–120 MGD, serving a population of 26,651–523,765, covering ≈83.6% of the county population. In Onondaga County, we sampled 7 treatment plants, each with a capacity of 0.99–84 MGD, serving a population of 3,272–242,377, covering ≈85.7% of the county population. In Jefferson County we sampled 5 treatment plants, each with a capacity 0.06–15.9 MGD, serving a population of 81–26,270, covering ≈46.7% of the county population. The New York State Wastewater Surveillance Network does not routinely collect treatment plant flow data.

### Wastewater Sampling and Processing

Plant operators collected 24-hour flow-weighted composite influent (before treatment) wastewater samples 1 or 2 times each week at wastewater plants (WWTPs) participating in the state network (*19*). In Erie County, the samples were processed at an environmental engineering laboratory located at the University at Buffalo. Specifically, we processed 9.75-mL wastewater samples using Nanotrap Microbiome A particles (Ceres Nanosciences, https://www.ceresnano.com), then performed nucleic acid extraction with MagMAX viral/pathogen nucleic acid isolation kits (Thermo Fisher Scientific, https://www.thermofisher.com). We eluted nucleic acids in 50 µL of MagMAX elution buffer. We automated both steps on the KingFisher Apex purification system (Thermo Fisher Scientific), as described previously (*20*). Samples from Jefferson, Onondaga, and Westchester counties were shipped overnight on wet ice to Quadrant Biosciences (Syracuse, NY, USA). In brief, we ultracentrifuged 20-mL samples at 150,000 × *g* at 40°C on a Sorvall WX Ultra series (Thermo Fisher Scientific) with a Sorvall SureSpin 630 (6 × 36 mL) with a sucrose cushion (*21*). We resuspended pellets in 200 μL 1X PBS and transferred them to 1.7-mL microcentrifuge tubes stored at −20°C for <24 h until nucleic acid extraction.

### Molecular Testing

We extracted total nucleic acids from pellets using the AllPrep PowerViral DNA/RNA Kit (QIAGEN, https://www.qiagen.com). We eluted nucleic acids in 50 μL elution buffer and synthesized cDNA using the QuantiTect reverse transcription kit (QIAGEN). We quantified total nucleic acid and cDNA by quantitative reverse transcription PCR and quantitative PCR. We used QuantStudio 3 or QuantStudio 5 (Thermo Fisher) real-time PCR systems for all quantitative PCR reactions. We detected and quantified norovirus nucleic acid with 5 µL of the extracted nucleic acid added to 45 µL of norovirus master mix, containing 900 µM of primers and 250 µM of probes with the QIAcuity microfluidic digital PCR instrument (QIAGEN), according to the manufacturer’s specifications. The cycling profile was 1 × 50°C for 40 minutes, 1 × 95°C for 2 minutes, and 40 × 95°C for 5 seconds followed by 60°C for 30 seconds. We performed an internal assay validation, including limit of detection, specificity, and reproducibility, before implementation of the pilot study. We empirically determined the limit of detection as 5 gene copies/μL of reaction and quantified mean norovirus GI and GII concentrations per microliter of reaction. The specificity panel for digital PCR detected no cross-reactivity for any of the 23 targets tested (*22*). We included negative and positive controls on all plates. We used norovirus GI- and GII-specific primers and probes implemented for a previously described digital PCR assay for the detection and quantification of hepatitis A and norovirus (*23*) (Appendix Table 1).

### Hospitalization Data

We retrieved patient-level acute gastroenteritis hospitalization data from the Statewide Planning and Research Cooperative System that captures hospitalizations from all hospitals in New York. We considered 2 types of hospitalizations: those specifically for norovirus-confirmed acute gastroenteritis and those related to acute gastroenteritis from any cause. We used specific codes for gastroenteritis from the International Classification of Diseases, 10th Revision, Clinical Modification (*24*) (Appendix Table 2), to identify hospital admissions for acute gastroenteritis (AGE). We removed patient records with missing address information, geocoded addresses to latitude and longitude, and aggregated hospitalizations to county level.

### Wastewater Measures

**Figure 15:**
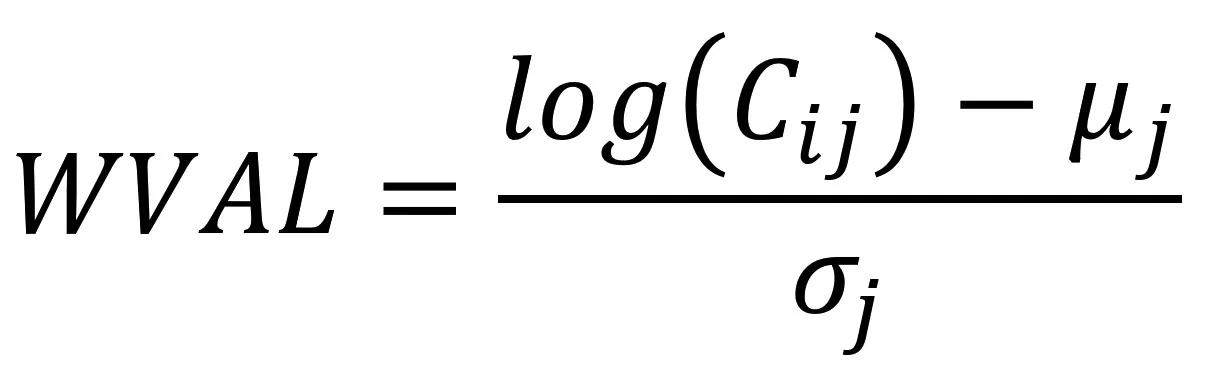
We calculated the wastewater activity level (WVAL) for each wastewater sample *i* in wastewater treatment plant *j* as the concentration of norovirus RNA in a sample (*C_ij_*) relative to the mean of norovirus concentrations in the treatment plant (*μ_j_*) and the standard deviation of norovirus concentrations in the treatment plant (*σ_j_*) using this equation.

We considered 2 different measures of the amount of norovirus in wastewater. First, we used the concentration of norovirus RNA for each genogroup per unit of wastewater. Second, we calculated the norovirus wastewater activity level (WVAL) as a continuous metric with equation 1. WVAL is a metric established by the Centers for Disease Control and Prevention (CDC) to monitor the level of virus in wastewater across diverse geographies and laboratory testing methodologies (*25*). For both approaches, we did not normalize the amount of norovirus in wastewater to the flow of the WWTP. We calculated the wastewater activity level (WVAL) for each wastewater sample *i* in wastewater treatment plant *j* as the concentration of norovirus RNA in a sample (*C_ij_*) relative to the mean of norovirus concentrations in the treatment plant (*μ_j_*) and the standard deviation of norovirus concentrations in the treatment plant (*σ_j_*) using this equation (Figure 15):

### Correlation of Norovirus in Wastewater and Acute Gastroenteritis Hospitalization

We considered 2 different outcomes of AGE hospitalizations: all-cause AGE hospitalizations and norovirus-specific AGE hospitalizations. For each outcome we conducted 2 types of analyses to estimate the correlation of norovirus in wastewater and AGE hospitalizations. First, we compared the mean monthly concentration or WVAL of norovirus in wastewater to the monthly incidence of AGE hospitalizations as previously described (*11*). Second, we compared weekly wastewater norovirus concentrations or WVALs with AGE hospitalizations. We conducted those analyses for both GI and GII norovirus concentrations. We used a Spearman rank correlation coefficient (ρ) to assess relationships between norovirus concentrations in wastewater and AGE hospitalizations. We also conducted cross-correlation analysis to identify potential time lags between wastewater norovirus concentrations and AGE hospitalizations.

To create county-level measures of norovirus concentration and WVAL, we estimated a population-weighted mean concentration of norovirus within a county by weighting each weekly or monthly norovirus concentration by the proportion of the county population served by each WWTP. We then regressed the monthly correlation coefficient between norovirus concentrations in wastewater and AGE hospitalizations to identify county-level factors that may have influenced the relationship. Last, using analysis of variance (ANOVA), we compared seasonal concentrations of GI and GII. We conducted all analyses using R version 4.4.1 (The R Project for Statistical Computing, http://www.r-project.org).

## Results

We analyzed 2,823 wastewater samples collected over 2 years. Detection rate for norovirus GI was 100% and for GII 92%–100% (Figure 1). During the study period, we observed 499 norovirus-specific AGE hospitalizations and 50,004 all-cause AGE hospitalizations.

### Quantification of Viral Load in Wastewater Samples

**Figure 2:**
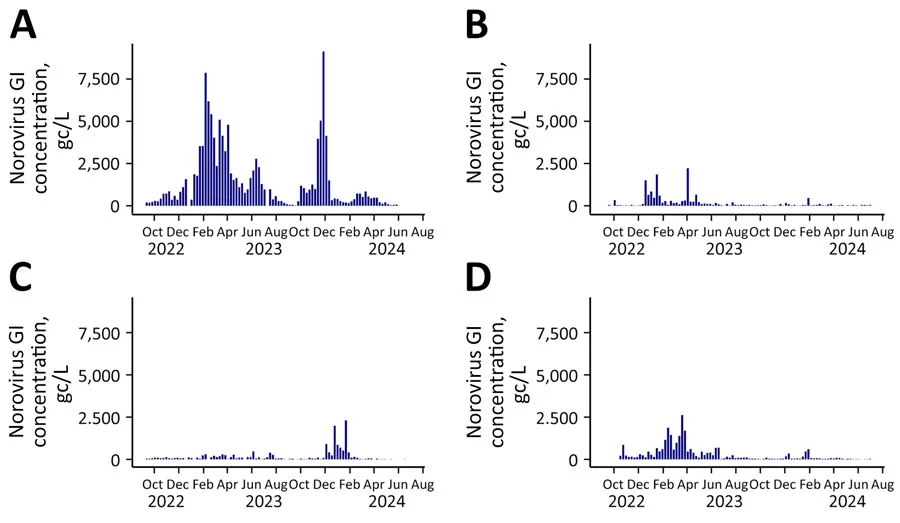
Population-weighted concentration of norovirus genotype I in wastewater over time by area in study of correlation between norovirus in wastewater with norovirus-attributable and general acute gastroenteritis-related hospitalizations, New York, USA, 2022–2024. A) Erie County. B) Jefferson County. C) Onondaga County. D) Westchester County. gc, gene copies.

**Figure 3:**
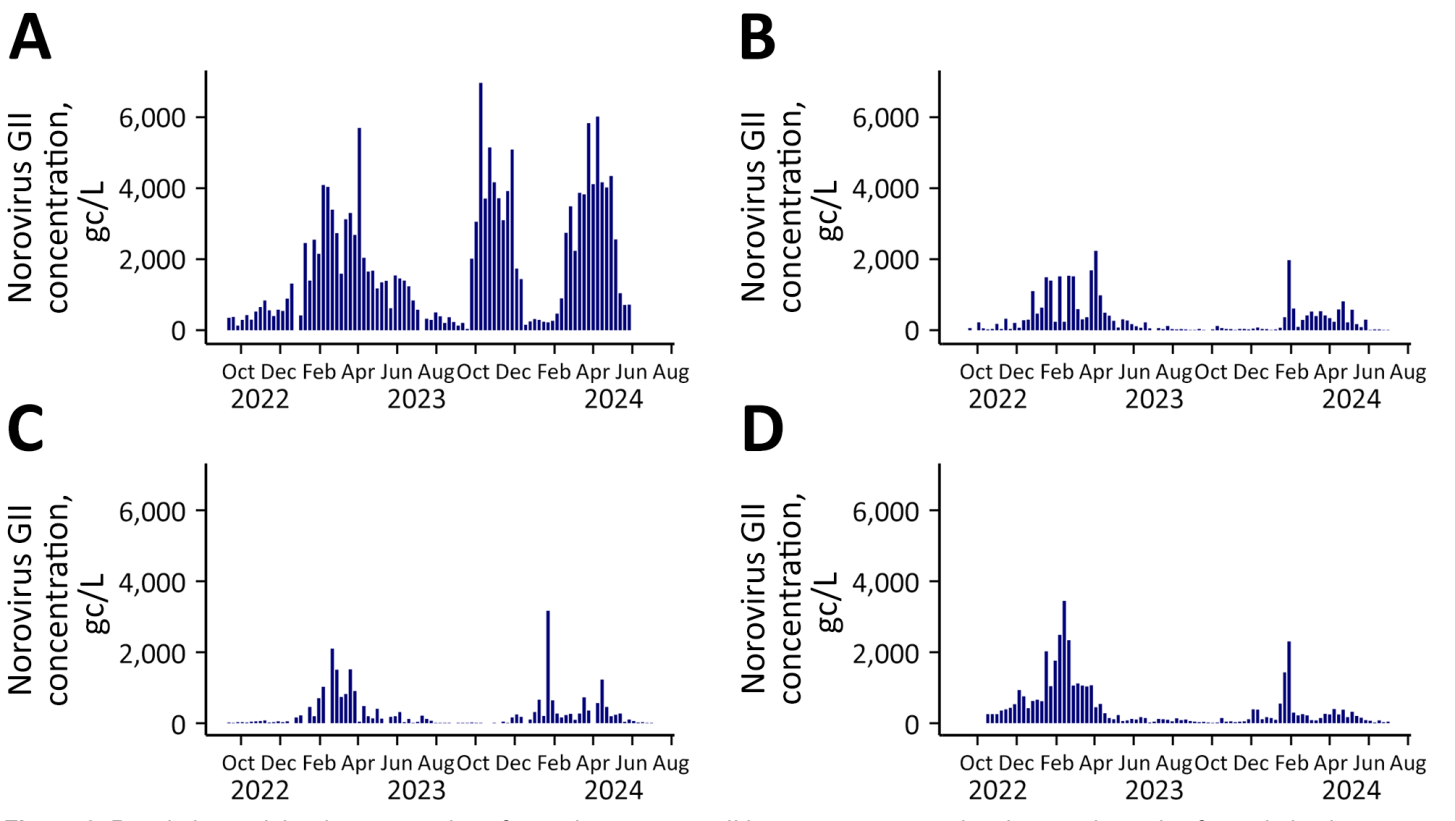
Population-weighted concentration of norovirus genotype II in wastewater over time by area in study of correlation between norovirus in wastewater with norovirus-attributable and general acute gastroenteritis-related hospitalizations, New York, USA, 2022–2024. A) Erie County. B) Jefferson County. C) Onondaga County. D) Westchester County. gc, gene copies.

**Figure 4:**
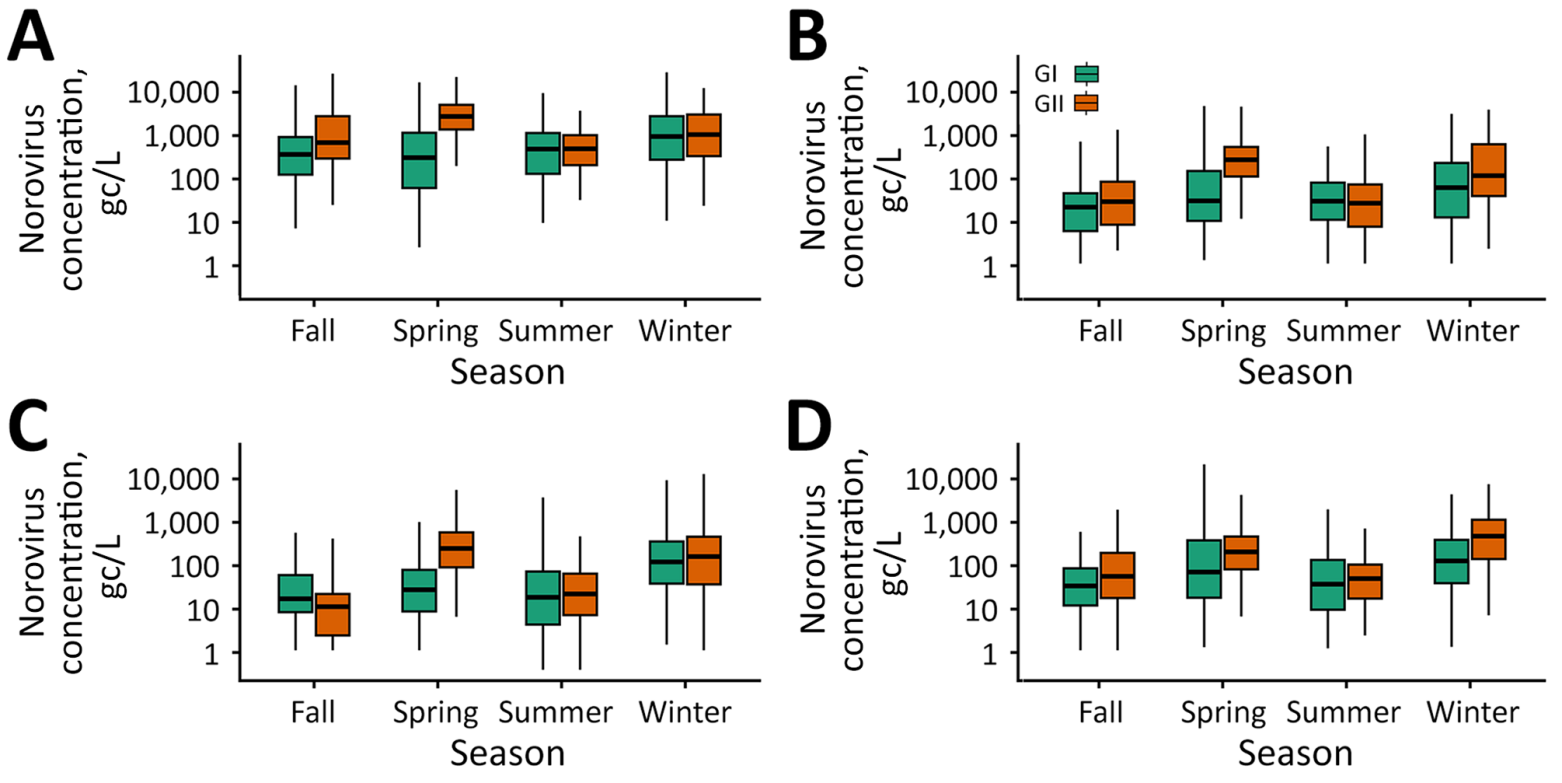
Distribution of norovirus recovered from wastewater by season in study of correlation between norovirus in wastewater with norovirus-attributable and general acute gastroenteritis-related hospitalizations, New York, USA, 2022–2024. A) Erie County. B) Jefferson County. C) Onondaga County. D) Westchester County. Horizontal lines within box plots indicate median concentrations, box tops and bottoms indicate interquartile range, and vertical bars indicate the full range of the data. gc, gene copies.

Weekly concentrations of norovirus GI and GII in wastewater samples aggregated to the county level revealed strong seasonal trends (Figures 2, 3). GI concentrations were highest in winter (December–February) and lowest in summer (June–August) (Figure 4). GII concentrations were highest in spring (March–May) and also lowest in summer (Figure 4). ANOVA of norovirus GI revealed that the differences in mean virus concentrations across seasons were statistically significant (*F*[3; 2,816] = 8.054; p<0.001). Tukey post-hoc tests showed significant differences between winter and fall (p<0.01), winter and spring (p<0.01), and winter and summer (p<0.001); we saw higher mean GI concentrations in winter in all comparisons. However, we found no significant differences between spring and fall, summer and fall, or summer and spring (p>0.05 for all). For norovirus GII, ANOVA result showed statistically significant differences in means across seasons (*F*[3; 2,816] = 34.86; p<0.0001). Tukey post-hoc tests indicated significant differences between spring and fall (p<0.001), summer and fall (p<0.001), summer and spring (p<0.001), winter and spring (p<0.001), and winter and summer (p<0.001). However, we found no significant difference between winter and fall (p = 0.3385). Erie County had an additional surge in norovirus GII wastewater levels during October–December 2023 that we did not observe in other counties (Figure 3); Erie County also had the highest concentrations of both GI and GII norovirus.

### Wastewater Norovirus Activity Level 

**Figure 5:**
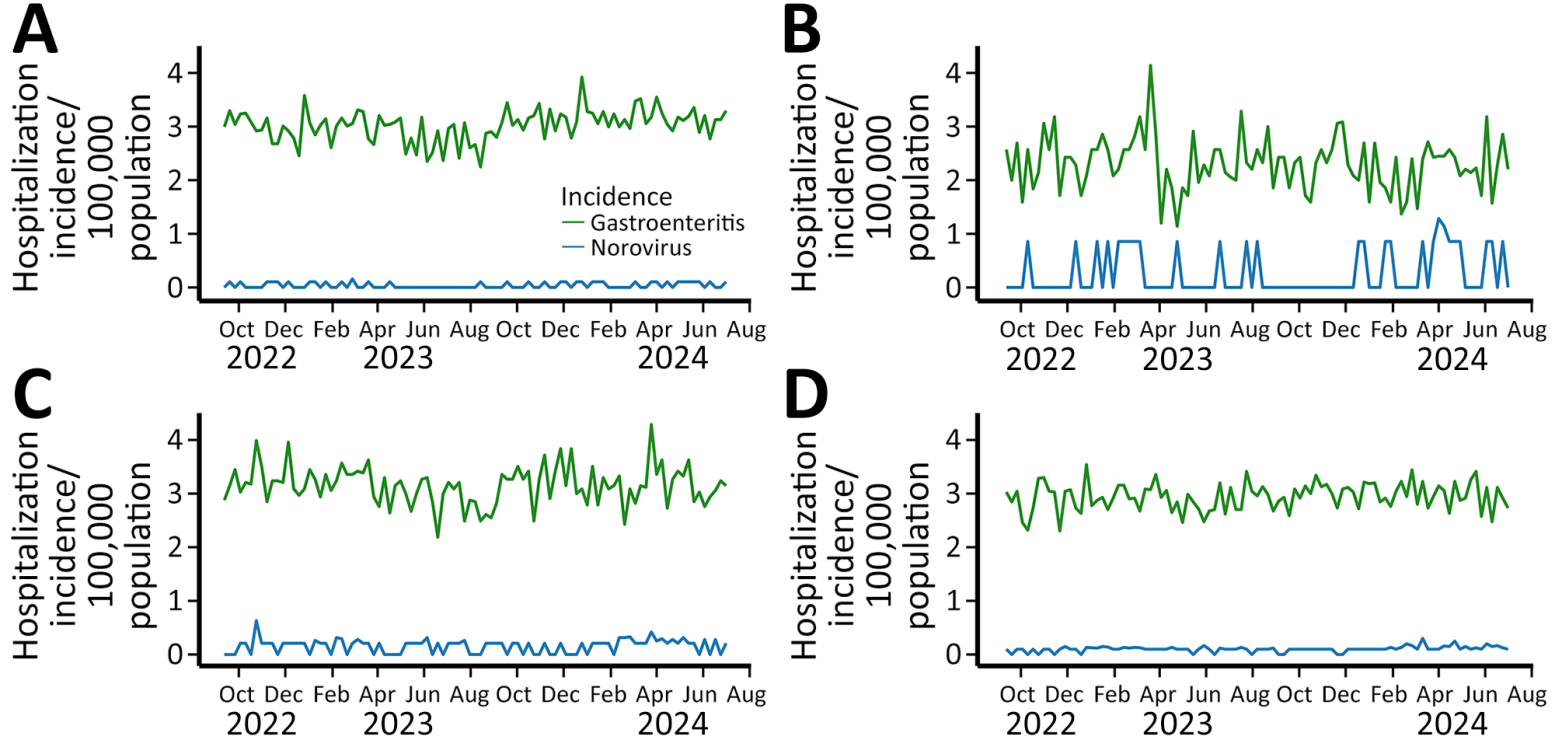
Incidence of all-cause and norovirus-specific hospitalizations for acute gastroenteritis over time in study of correlation between norovirus in wastewater with norovirus-attributable and general acute gastroenteritis-related hospitalizations, New York, USA, 2022–2024. A) Erie County. B) Jefferson County. C) Onondaga County. D) Westchester County.

In all the counties, both norovirus GI and GII WVAL showed seasonal trends similar to those observed with GI and GII norovirus wastewater trends (Figure 5). Across the counties, GI and GII WVAL were relatively low (mean values <2). A 1-way ANOVA showed that weighted WVAL GII differed significantly by season (*F*[3; 796] = 90.33; p<0.001). Similarly, weighted WVAL GI differed significantly by season (*F*[3; 796] = 23.9; p<0.001). Tukey post-hoc test showed noticeable seasonal structuring for weighted WVAL GII. Winter and spring showed significantly higher values compared with summer and fall. In contrast, weighted WVAL GI revealed a modest seasonal pattern of a visible winter peak and relatively similar values for spring, summer, and fall. Temporal trends of norovirus-specific and all-cause AGE hospitalization incidence with GI and GII WVAL showed no notable similarities (Appendix Figure 1).

### Temporal and Spatial Distribution of AGE Hospitalizations

**Figure 6:**
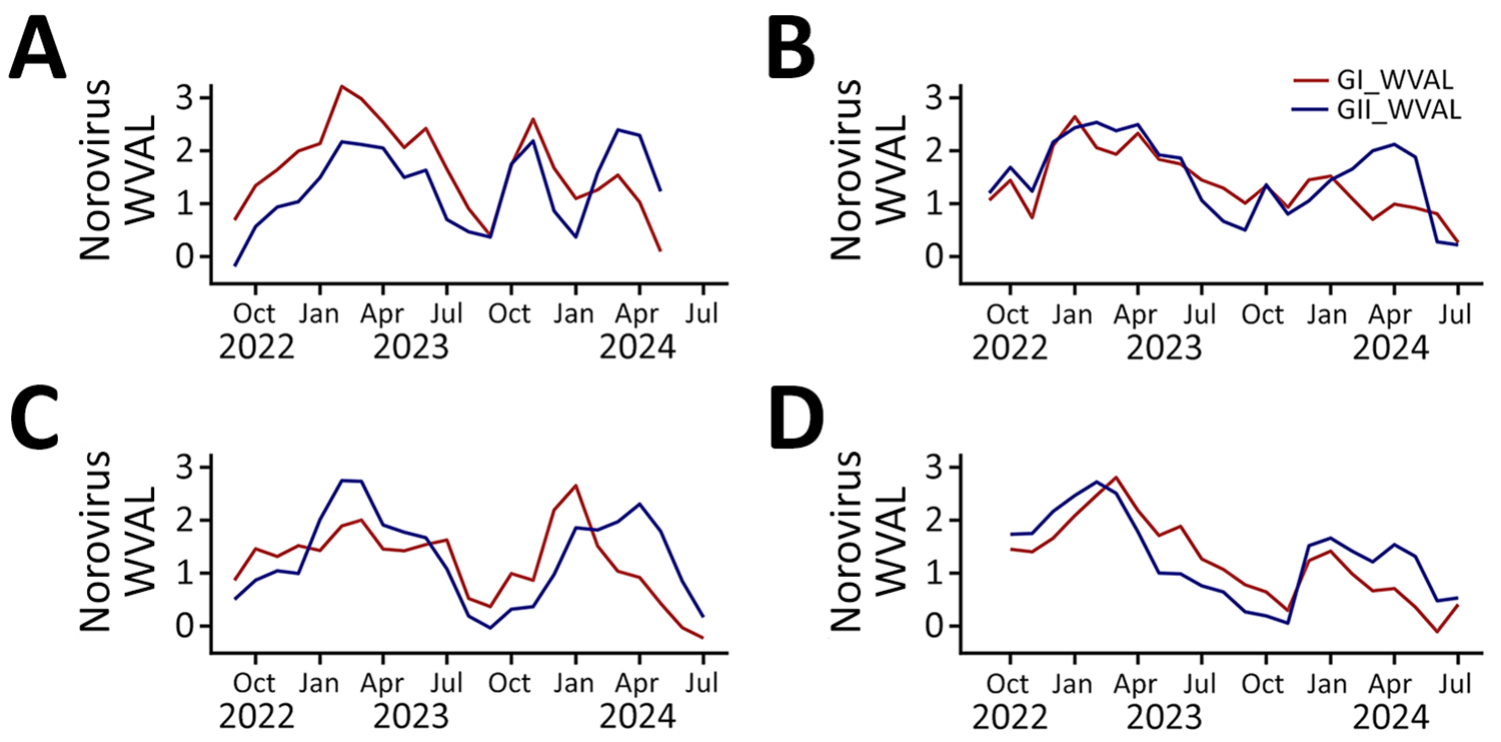
Relative abundance of norovirus in wastewater in study of correlation between norovirus in wastewater with norovirus-attributable and general acute gastroenteritis–related hospitalizations, New York, USA, 2022–2024. Abundance was measured as wastewater activity level, which increased in the winter months and decreased in the summer months. A) Erie County. B) Jefferson County. C) Onondaga County. D) Westchester County. WVAL, norovirus wastewater activity level.

Weekly hospitalizations across the participating counties revealed both the temporal and spatial patterns in norovirus and all-cause AGE hospitalization incidence; incidence of norovirus-specific hospitalizations was slightly higher in the winter and spring (Figure 6). ANOVA indicated statistically significant difference in seasonal norovirus hospitalization incidence (*F*[3; 376] = 4.666; p<0.01). Tukey post-hoc analysis showed significant differences in norovirus hospitalization incidence between spring and fall (p<0.01) and between winter and fall (p<0.01); we observed no significant differences between other season pairs (p>0.05). The incidence of all-cause AGE hospitalizations remained relatively stable over the study period (p>0.05); seasonal mean incidence was 2.87 for spring, 2.87 for winter, 2.91 for fall, and 2.74, for summer. Onondaga County had the highest mean incidence of all-cause AGE hospitalizations (3.15/100,000 population), followed closely by Erie County (3.02/100,000 population), then Westchester County (2.94/100,000 population) and Jefferson County (2.31/100,000 population). Jefferson County had the highest norovirus AGE mean incidence (0.251/100,000 population); Erie County had the lowest norovirus AGE mean incidence (0.039/100,000 population). Mean incidence of norovirus hospitalizations for Onondaga County was 0.159/100,000 population and for Westchester County was 0.103/100,000 population.

### GI and GII Concentrations and Norovirus and All-Cause AGE Hospitalizations Incidence

**Figure 7:**
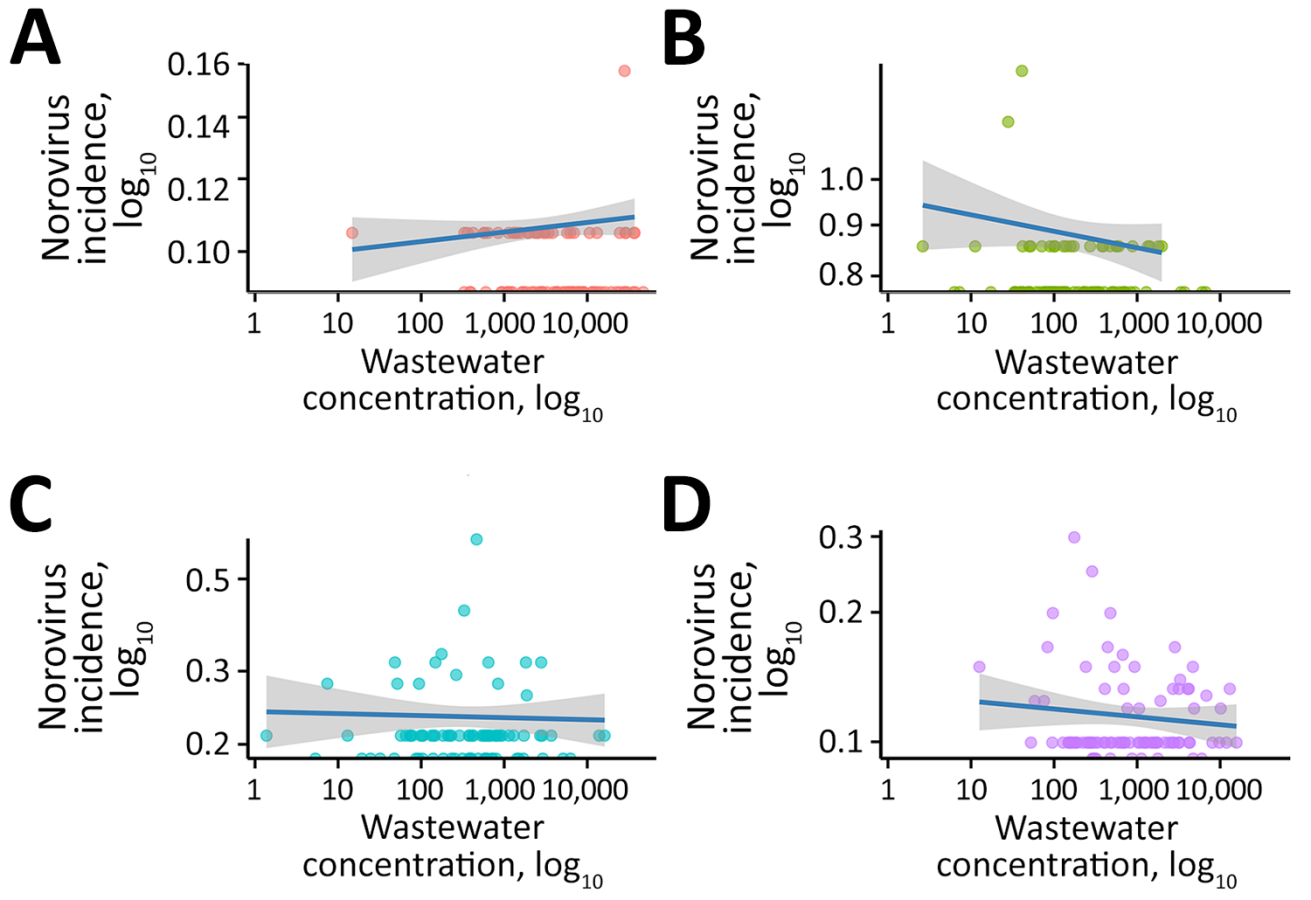
Correlations between population-weighted concentration of norovirus genotype I in wastewater and norovirus-specific incidence of acute gastroenteritis–related hospitalizations in study of correlation between norovirus in wastewater with norovirus-attributable and general acute gastroenteritis–related hospitalizations, New York, USA, 2022–2024. Blue line represents linear regression fit and gray shading indicates 95% CIs. A) Erie County; Spearman rank correlation coefficient (ρ) = −0.18. B) Jefferson County; ρ= 0.06. C) Onondaga County; ρ = 0. D) Westchester County; ρ = −0.1.

**Figure 8:**
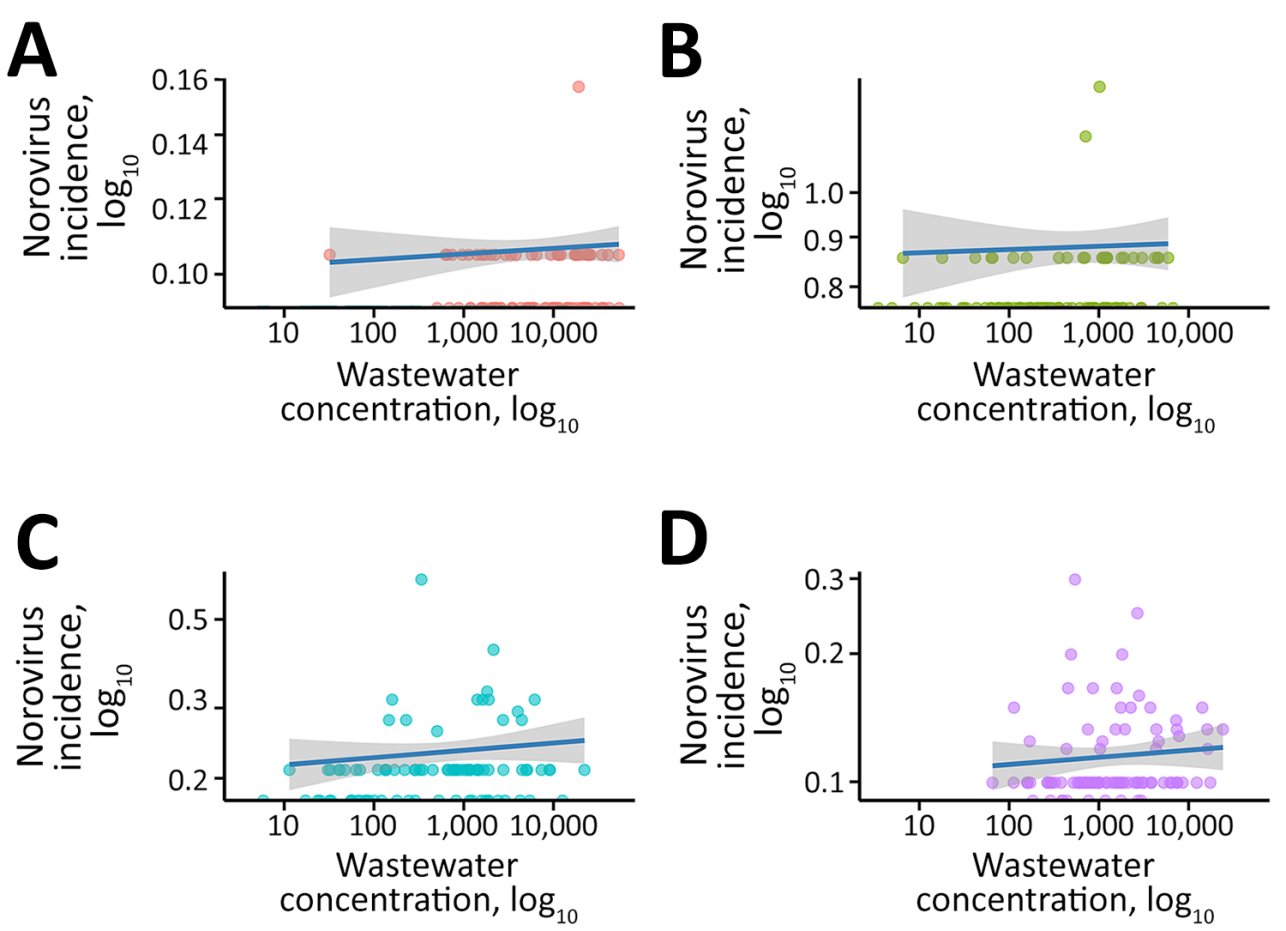
Correlations between population-weighted concentration of norovirus genotype II in wastewater and norovirus-specific incidence of acute gastroenteritis–related hospitalizations in study of correlation between norovirus in wastewater with norovirus-attributable and general acute gastroenteritis–related hospitalizations, New York, USA, 2022–2024. Blue line represents linear regression fit and gray shading indicates 95% CIs. A) Erie County; Spearman rank correlation coefficient (ρ) = −0.03. B) Jefferson County; ρ = 0.26. C) Onondaga County; ρ = 0.28. D) Westchester County; ρ = 0.23.

**Figure 9:**
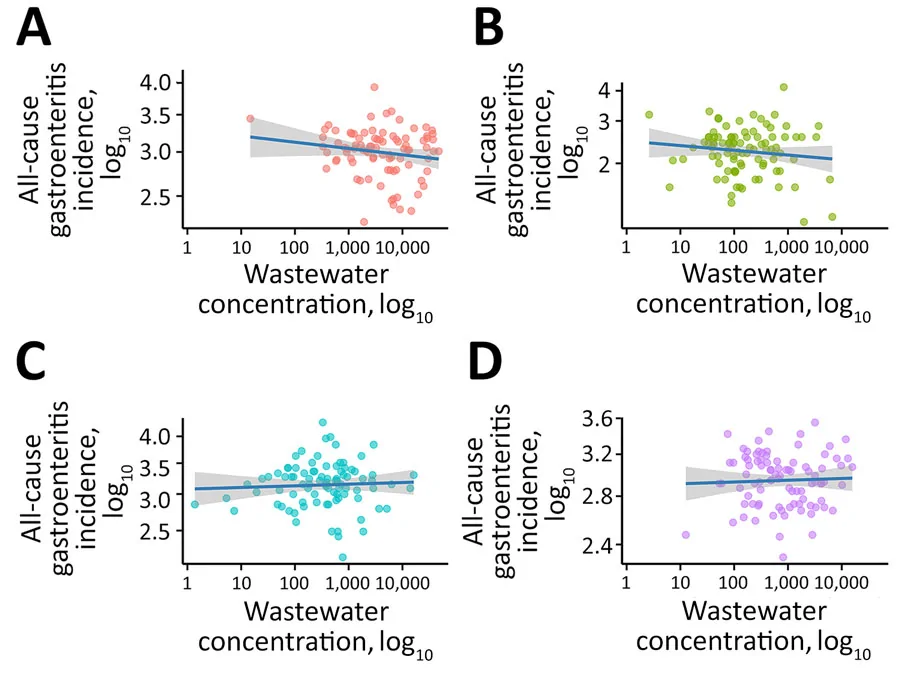
Correlations between population-weighted concentration of norovirus genotype I in wastewater and incidence of all-cause acute gastroenteritis–related hospitalizations in study of correlation between norovirus in wastewater with norovirus-attributable and general acute gastroenteritis–related hospitalizations, New York, USA, 2022–2024. Blue line represents linear regression fit and gray shading indicates 95% CIs. A) Erie County; Spearman rank correlation coefficient (ρ) = −0.12. B) Jefferson County; ρ = −0.07. C) Onondaga County; ρ = 0.03. D) Westchester County; ρ = −0.04.

**Figure 10:**
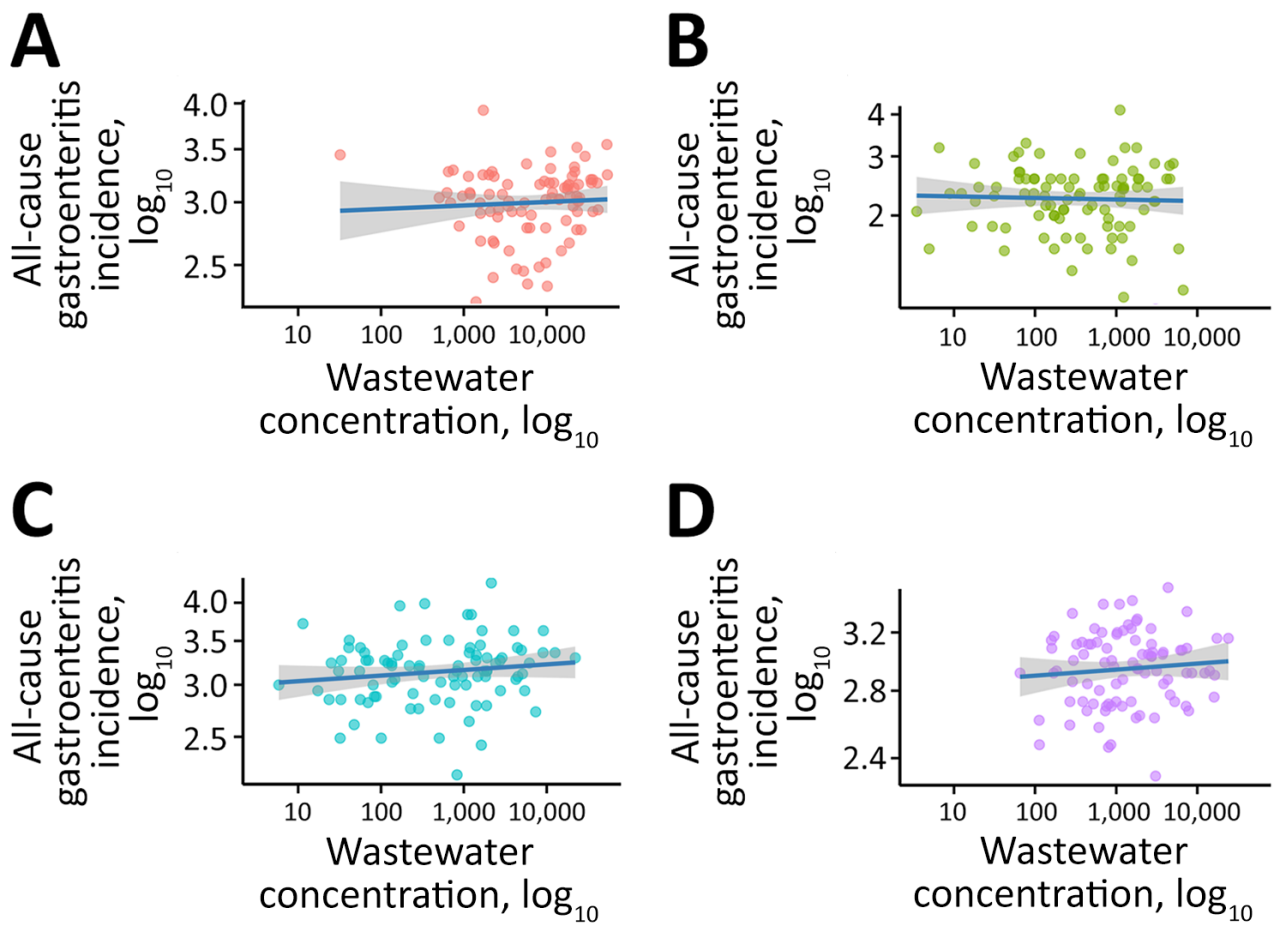
Correlations between population-weighted concentration of norovirus genotype II in wastewater and incidence of all-cause acute gastroenteritis–related hospitalizations in study of correlation between norovirus in wastewater with norovirus-attributable and general acute gastroenteritis–related hospitalizations, New York, USA, 2022–2024. Blue line represents linear regression fit and gray shading indicates 95% CIs. A) Erie County; Spearman rank correlation coefficient (ρ) = 0.12. B) Jefferson County; ρ = 0.02. C) Onondaga County; ρ = 0.17. D) Westchester County; ρ = 0.07.

We determined weekly correlations between population-weighted GI and GII concentrations and the incidence of norovirus hospitalizations (Figures 7, 8) or all-cause AGE hospitalizations for the 4 counties (Figures 9, 10). Correlation between population-weighted GI concentrations with incidence of norovirus hospitalizations was −0.18 to 0.06 (Figure 7) and for all-cause AGE hospitalizations was −0.12 to 0.03 (Figure 9). Correlations between population-weighted GII concentrations and incidence of norovirus hospitalizations showed moderate positive relationship in 3 counties; Spearman ρ was 0.23–0.28 (Figure 8). Population-weighted GII concentrations and all-cause AGE were also weakly correlated; Spearman ρ was −0.02 to 0.17 (Figure 10). Monthly correlations between population-weighted GI concentrations and incidence of norovirus hospitalizations or all-cause AGE for the 4 counties showed no significant correlation, whereas correlations between population-weighted GII concentrations and incidence of norovirus hospitalizations and all-cause AGE hospitalizations improved in 3 counties (Appendix Figure 2).

**Figure 11:**
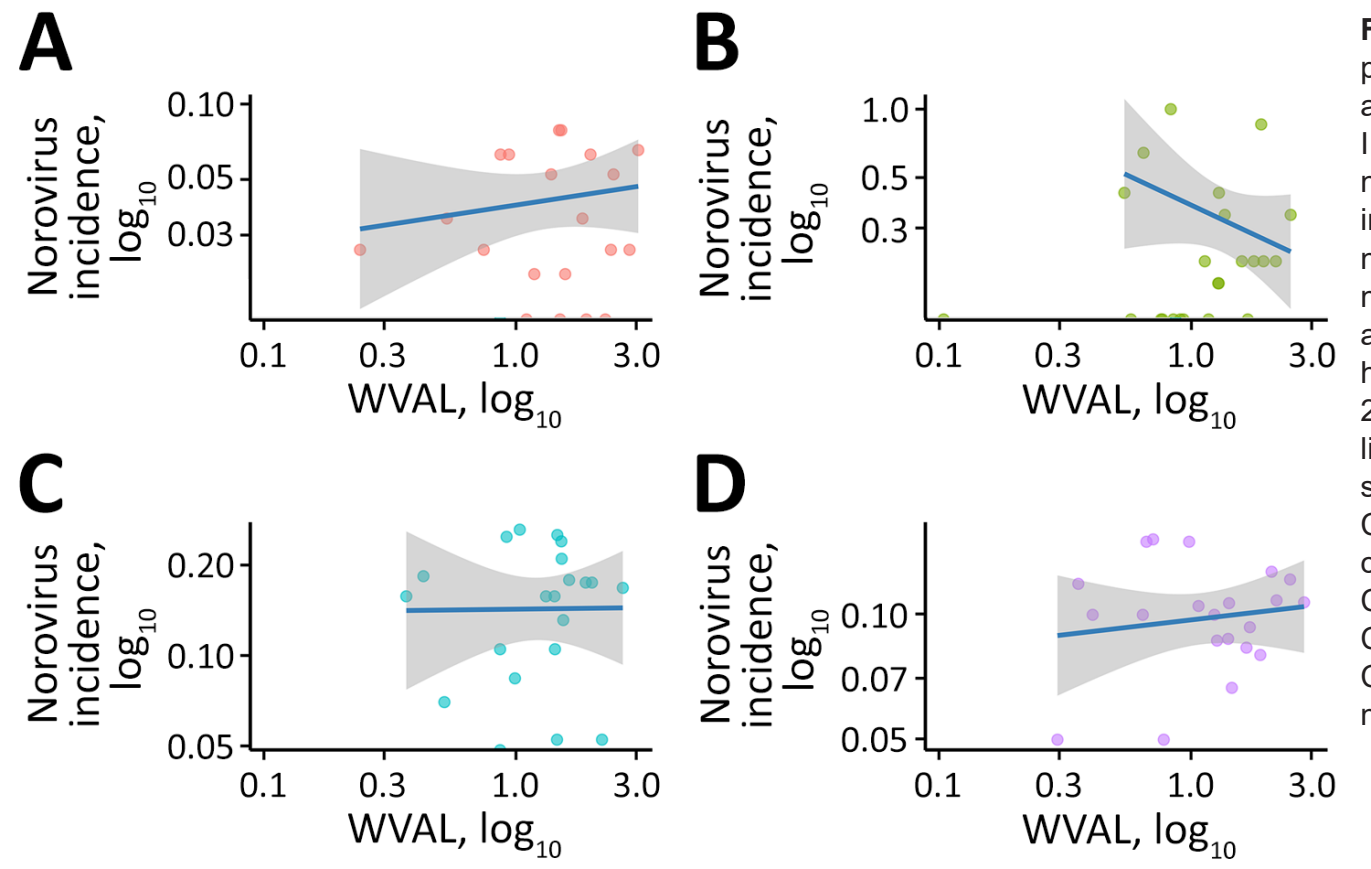
Correlations between population-weighted wastewater activity level of norovirus genotype I in wastewater and incidence of norovirus-specific hospitalizations in study of correlation between norovirus in wastewater with norovirus-attributable and general acute gastroenteritis–related hospitalizations, New York, USA, 2022–2024. Blue line represents linear regression fit and gray shading indicates 95% CIs. A) Erie County; Spearman rank correlation coefficient (ρ) = −0.12. B) Jefferson County; ρ = 0.3. C) Onondaga County; ρ= 0.07. D) Westchester County; ρ = −0.09. WVAL, norovirus wastewater activity level.

**Figure 12:**
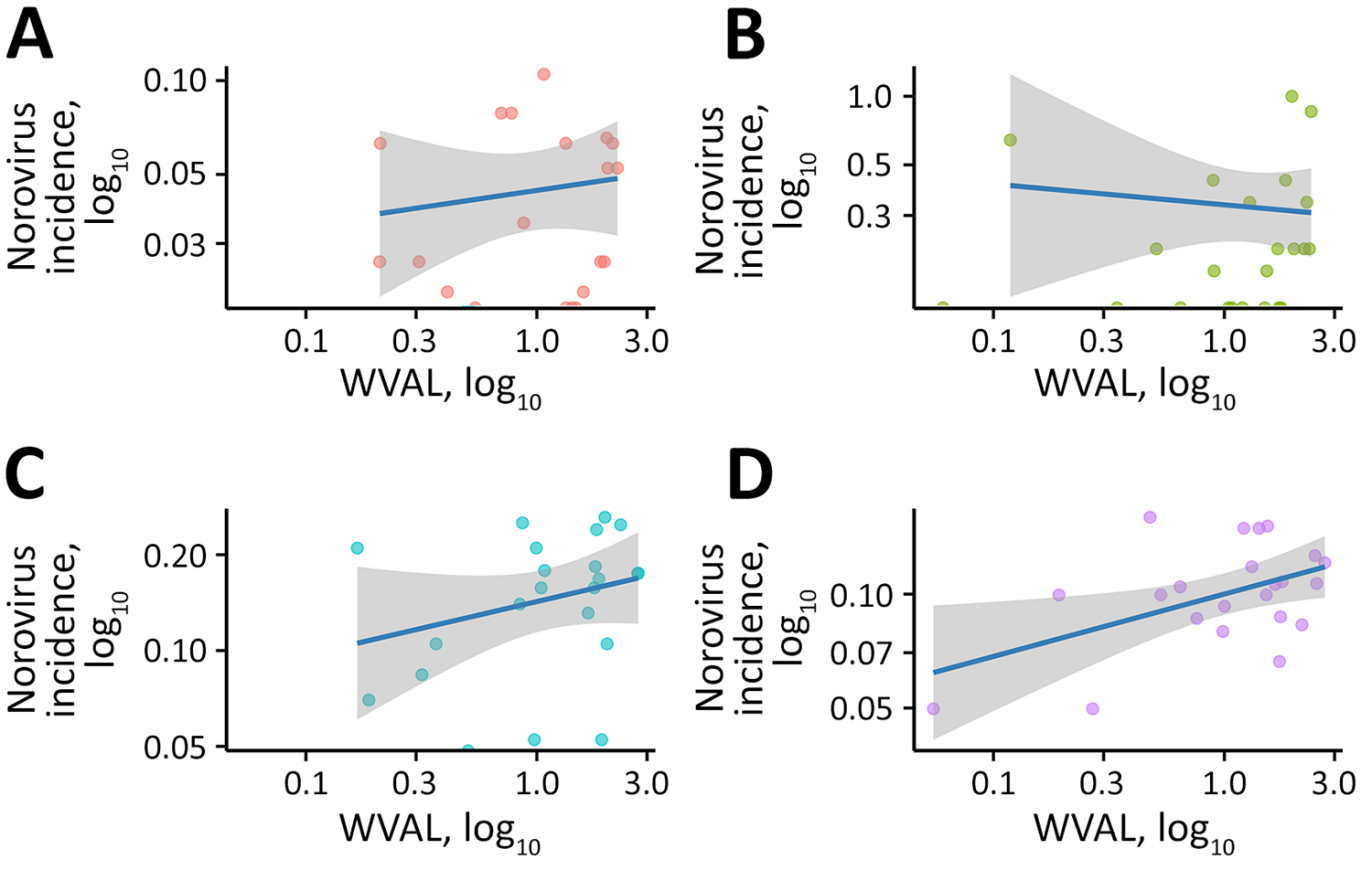
Correlations between population-weighted wastewater activity level of norovirus genotype II in wastewater and incidence of norovirus-specific hospitalizations in study of correlation between norovirus in wastewater with norovirus-attributable and general acute gastroenteritis–related hospitalizations, New York, USA, 2022–2024. Blue line represents linear regression fit and gray shading indicates 95% CIs. A) Erie County; Spearman rank correlation coefficient (ρ) = 0.05. B) Jefferson County; ρ = 0.38. C) Onondaga County; ρ = 0.32. D) Westchester County; ρ = 0.29. WVAL, norovirus wastewater activity level.

**Figure 13:**
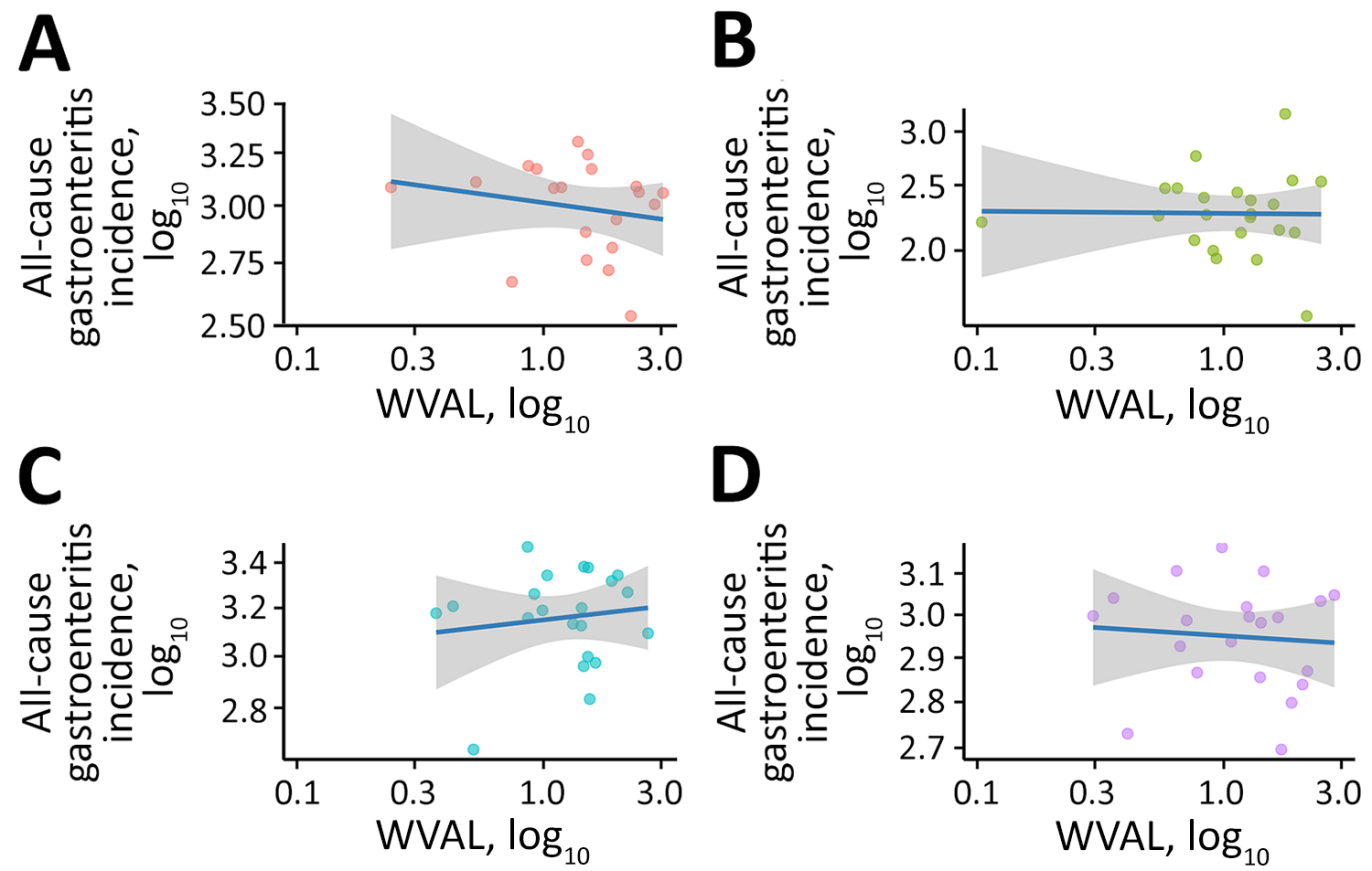
Correlations between population-weighted wastewater activity level of norovirus genotype I in wastewater and incidence of all-cause acute gastroenteritis–related hospitalizations in study of correlation between norovirus in wastewater with norovirus-attributable and general acute gastroenteritis–related hospitalizations, New York, USA, 2022–2024. Blue line represents linear regression fit and gray shading indicates 95% CIs. A) Erie County; Spearman rank correlation coefficient (ρ) = −0.35. B) Jefferson County; ρ = −0.03. C) Onondaga County; ρ = 0.07. D) Westchester County; ρ = −0.1. WVAL, norovirus wastewater activity level.

**Figure 14:**
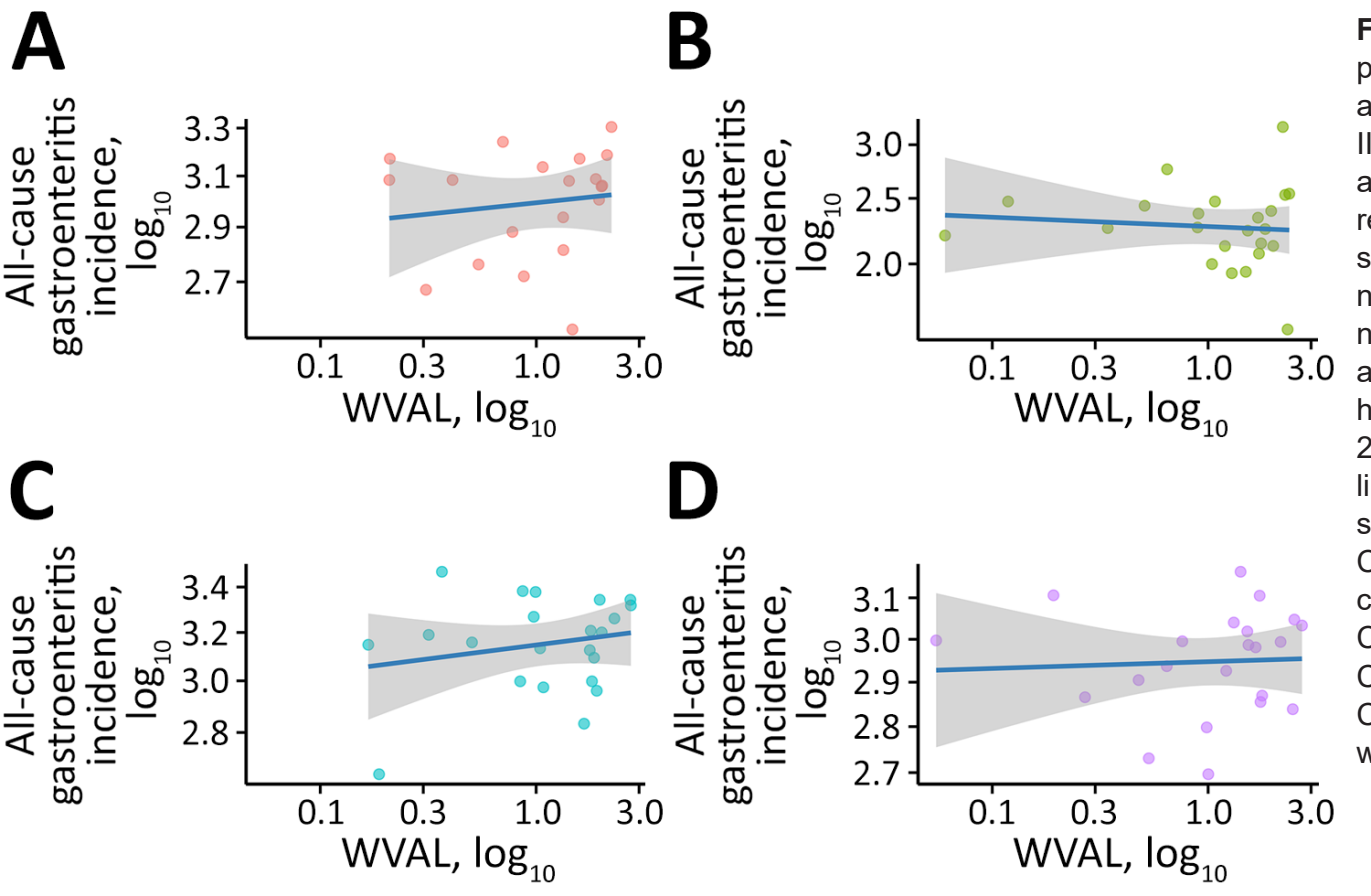
Correlations between population-weighted wastewater activity level of norovirus genotype II in wastewater and incidence of all-cause acute gastroenteritis–related hospitalizations in study of correlation between norovirus in wastewater with norovirus-attributable and general acute gastroenteritis–related hospitalizations, New York, USA, 2022–2024. Blue line represents linear regression fit and gray shading indicates 95% CIs. A) Erie County; Spearman rank correlation coefficient (ρ) = 0.14. B) Jefferson County; ρ = −0.03. C) Onondaga County; ρ = 0.13. D) Westchester County; ρ = 0.14. WVAL, norovirus wastewater activity level.

Monthly correlations between norovirus WVAL and the incidence of norovirus-specific and all-cause AGE incidence showed a similar relationship to correlations between norovirus concentrations and incidence (Figures 11–14). County-specific correlations revealed no significant correlations between monthly norovirus incidence (Figure 11) and monthly all-cause AGE hospitalization incidence (Figure 13) with GI WVAL. County-specific association between norovirus incidence and GII WVAL indicated moderate correlation in Jefferson, Onondaga, and Westchester counties (Spearman ρ 0.29–0.38) (Figure 12); correlation with all-cause AGE hospitalization incidence was low (ρ<0.2) for all 4 counties (Figure 14). However, we observed no significant correlations between weekly norovirus GI or GII WVAL and norovirus incidence and gastroenteritis incidence (Appendix Figure 3).

## Discussion

We consistently detected GI and GII norovirus in wastewater collected during October 2022–July 2024 across 4 counties in New York: GI in 100% of samples and GII in 92%–100% of samples. We observed strong seasonal trends, with higher concentrations of both genogroups in winter and spring and lower concentrations in summer, aligning with the established seasonality of norovirus hospitalizations (*26*,*27*). Hospitalizations showed significant seasonal differences (Figure 6), with higher incidence in winter and spring, which we did not observe for all-cause AGE hospitalizations. Because incidence of norovirus-specific AGE was low, it is difficult to see those differences. Correlations between wastewater concentrations and hospitalization incidence were generally weak to moderate (−0.18 to 0.28). Neither monthly nor weekly correlations were significantly stronger with the WVAL metric.

Our findings are consistent with previous studies that reported weak to moderate positive correlations between norovirus wastewater measures and clinical measures. A study of county data in Michigan, United States (*12*), showed human norovirus GII wastewater levels leading total weekly gastrointestinal illness–related emergency room visits by 2 weeks; correlations were ρ = −0.20 to 0.81. In British Columbia (*14*), norovirus levels in wastewater and weekly norovirus outbreaks were also positively correlated; Kendall τ correlation was 0.21–0.36, depending on normalization method. A linear regression analysis of data in England (*15*) showed a significant linear relationship between norovirus levels in wastewater and weekly cases (R^2^ = 0.614). Last, a systematic and meta-analysis study (*11*) of norovirus wastewater correlations with cases and outbreaks reported associations of −0.03 to 0.44. However, difference in scale of spatial aggregation of wastewater data, such as nationwide aggregation (*13*), might suggest that improved correlations are a product of the modifiable area unit problem, whereas ecologic studies are subject to risk masking at regional or higher spatial aggregation (*28*). Large-scale spatial aggregation can affect risk perception and association and is unreliable for local epidemiologic interventions. Similarly, correlations between wastewater and clinical data might vary because of fundamental differences across case, hospitalization, and outbreak data. Also, wastewater data normalization methods using wastewater flow rate in liters/day or population served by each WWTP might affect the relationship between norovirus levels in wastewater and clinical data.

The correlations we observed between norovirus GI and GII in wastewater and hospitalizations were lower than those observed in WBE studies of other pathogens (*29**–**31*). The observed seasonality, with peaks in winter and spring, corroborates established norovirus transmission patterns in temperate regions (*26*,*27*). Higher correlations between hospitalizations and GII concentrations may reflect the dominance of GII strains in large-scale outbreaks (*16*). Weak to moderate correlations between wastewater concentrations (GI or GII) and hospitalization incidence suggest that although WBE can capture population-level norovirus trends, it might not directly predict hospitalizations. That finding is consistent with previous inferences that, although WBE is effective for detecting community-level disease circulation, WBE results might not directly correlate with clinical disease occurrence (*32*,*33*). Possible reasons include temporal misalignment, spatial mismatch, scale of aggregation, heterogeneity in pathogen shedding, testing and reporting bias, and environmental and technical noise in wastewater data. Because norovirus infections are often asymptomatic or self-limiting and reporting is not mandatory, cases are underreported and clinical data are underrepresented (*34*). In addition, the lack of strong correlation between wastewater and clinical data likely reflects the self-limiting nature of norovirus and the fact that norovirus cases are underreported in clinical data.

The higher concentrations of norovirus in wastewater from Erie County are likely a result of differences in laboratory processing methods; some samples from Erie County underwent magnetic bead processing. We did not find evidence of elevated numbers of hospitalizations in Erie County, which suggests that the laboratory method is the likely reason for the difference. We observed similar differences in the recovery of SARS-CoV-2 in wastewater with the different concentration methods.

A limitation of our study is that WBE as a surveillance method cannot attribute viral loads to specific persons, which limits its use for providing granular epidemiologic data and follow-up crucial to outbreak investigations (*35*). Second, norovirus-specific hospitalization data are likely an underestimate of norovirus-caused hospitalizations. All-cause AGE is overly broad, and the norovirus-specific AGE International Classification of Diseases codes require laboratory confirmation of norovirus; not all patients will necessarily be tested for all pathogens. Third, wastewater was sampled weekly; however, disease duration of norovirus is often shorter, which might result in a temporal mismatch of data that could obscure finer-scale temporal dynamics and weaken the observed associations between wastewater viral concentrations and hospitalization incidence. We attempted to address that limitation by using the 7-day moving average but found no significant temporal lags or leads. Fourth, our study used 2 different laboratory methods, which made comparison between Erie County and the others more difficult but suggests that the method used to process Erie County samples resulted in greater nucleic acid recovery. Fifth, hospitalizations were relatively rare across the study area and period. It is likely that not all patients who sought care for norovirus infection at a hospital were tested or received diagnosis. Last, the New York state wastewater surveillance network does not routinely collect flow data from participating WWTPs, which precludes any examination of whether normalizing the norovirus concentration in wastewater by flow could improve correlation with hospitalization data.

The results of this study reinforce the ability of WBE to monitor circulating norovirus and suggest that AGE hospitalizations are a small part of the norovirus public health burden. Contrary to its use for other pathogens, such as COVID-19 (*29*), WBE is unlikely to be useful for predicting norovirus hospitalizations. The moderate correlations observed in Erie County and during peak seasons suggest that WBE could be optimized to inform public health interventions, especially during peak seasons, but more evidence is needed. Distinguishing between GI and GII trends highlights the importance of genogroup-specific monitoring; the association of GII with severe outbreaks (*36*,*37*) could guide resource allocation for outbreak preparedness. In this capacity, WBE is useful for understanding which norovirus genotypes are circulating in a particular population.

Appendix Additional information from study of correlation between norovirus in wastewater with norovirus-attributable and general acute gastroenteritis–related hospitalizations, New York, USA, 2022–2024.
