# Supplementary material for: Correlation of Norovirus in Wastewater with Gastroenteritis-Related Hospitalizations, New York, USA, 2022–2024
Source: Emerg Infect Dis. 2026 Aug;32(Suppl 1):S80–92. doi: 10.3201/eid3213.260472 (PMC13585665; doi:10.3201/eid3213.260472)
Supplement: Supplementary file 1 [file 26-0472-Techapp-s1.pdf]

Article DOI: https://doi.org/10.3201/eid3213.260472

EID cannot ensure accessibility for supplementary materials supplied by authors. Readers who have difficulty accessing supplementary content should contact the authors for assistance.

Correlation of Norovirus in Wastewater with Gastroenteritis-Related Hospitalizations, New York, USA, 2022–2024

Appendix

Appendix Table 1. Norovirus GI and GII primers and probes*

Genogroup	Gene	Gene name	Sequence
NoV GI	F	QNIF4	CGC TGG ATG CGN TTC CAT
NoV GI	R	NV1LCR	CCT TAG ACG CCA TCA TCA TTT AC
NoV GI	P	TM9	[FAM]-TGG ACA GGA GAT CGC-[MGB-NFQ*]
NoV GII	F	QNIF2	ATG TTC AGR TGG ATG AGR TTC TCW GA
NoV GII	R	COG2R	TCG ACG CCA TCT TCA TTC ACA
NoV GII	P	QNIFs	[CY-5]-AGC ACG TGG GAG GGC GAT CG-[BHQ-3‡]

*GI, genotype I; GII, genotype II; NoV, norovirus.
†MGB-NFQ, minor groove binder-nonfluorescent quencher
‡BHQ, black hole quencher

Appendix Table 2. ICD-10-CM codes related to acute gastroenteritis

Classification	ICD-10 code
Norovirus	A08.11
Norovirus	A08.1
Acute gastrointestinal	A08.19
Acute gastrointestinal	K52
Acute gastrointestinal	A09
Acute gastrointestinal	J11.2
Acute gastrointestinal	J09.X3
Acute gastrointestinal	J10.2
Acute gastrointestinal	Y73
Acute gastrointestinal	A08.4
Acute gastrointestinal	K29
Acute gastrointestinal	T78.4
Acute gastrointestinal	B97.89
Acute gastrointestinal	T61
Acute gastrointestinal	T62
Acute gastrointestinal	T78.1
Acute gastrointestinal	P74.1
Acute gastrointestinal	E86.0

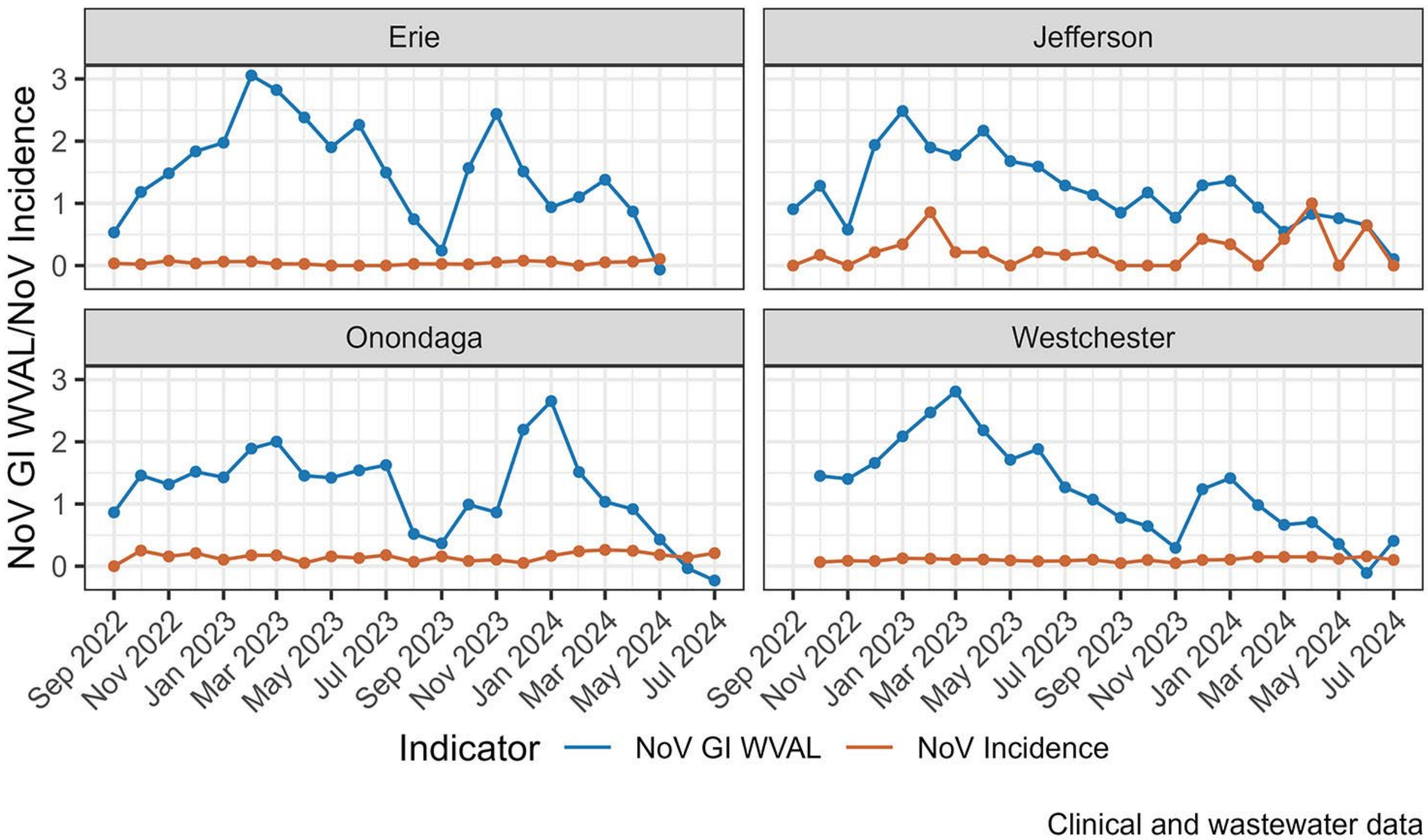

Appendix Figure 1. Temporal trends of (NoV) wastewater activity levels (WVAL) and hospitalization incidence across four New York State counties from September 2022 through July 2024. Monthly trends of population-weighted NoV GI WVAL (blue) overlaid with weekly NoV hospitalization incidence per 100,000 population (orange) by county.

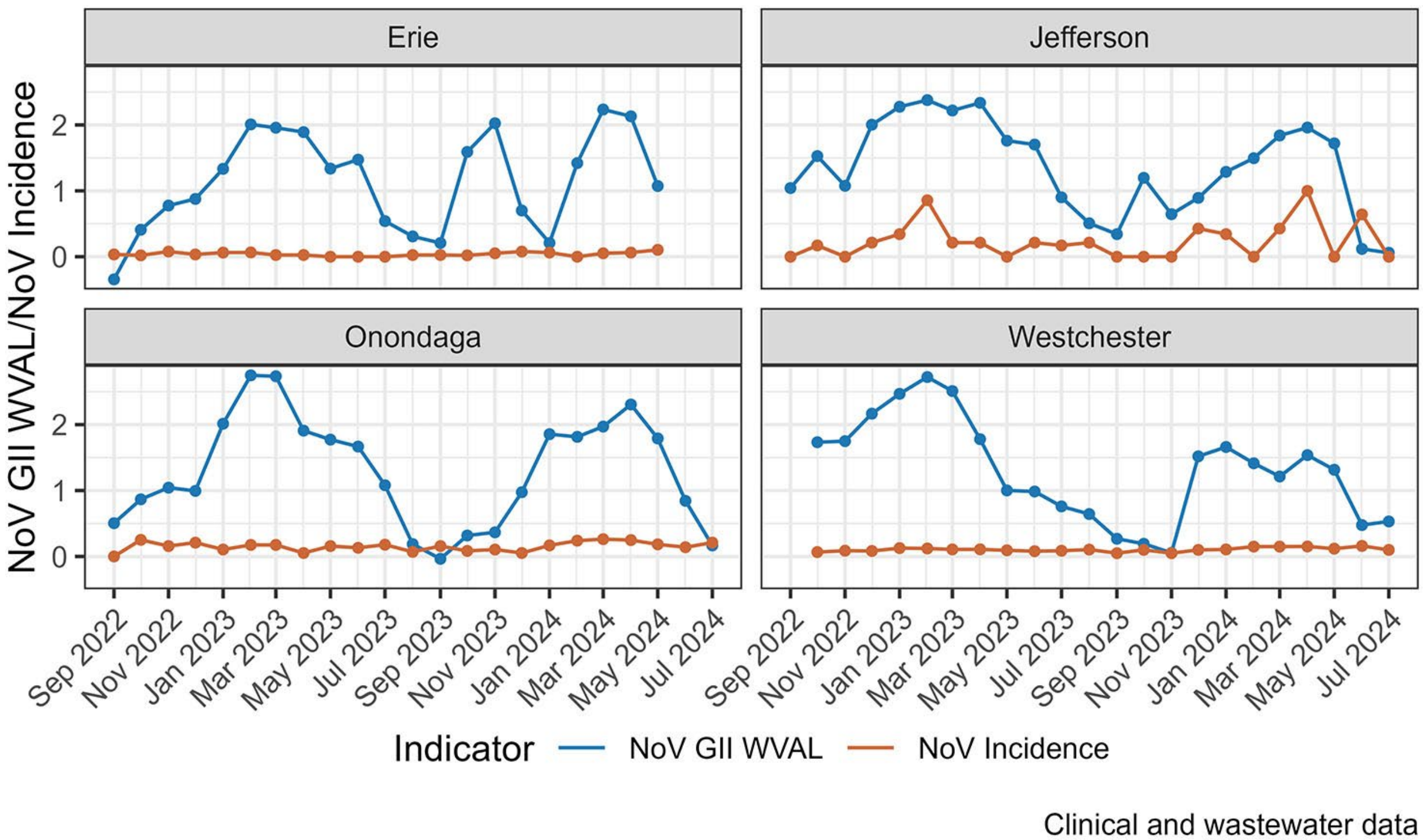

Appendix Figure 2. Temporal trends of norovirus (NoV) wastewater activity levels (WVAL) and hospitalization incidence across four New York State counties from September 2022 through July 2024. Monthly trends of population-weighted NoV GII WVAL (blue) overlaid with weekly NoV hospitalization incidence per 100,000 population (orange) by county.

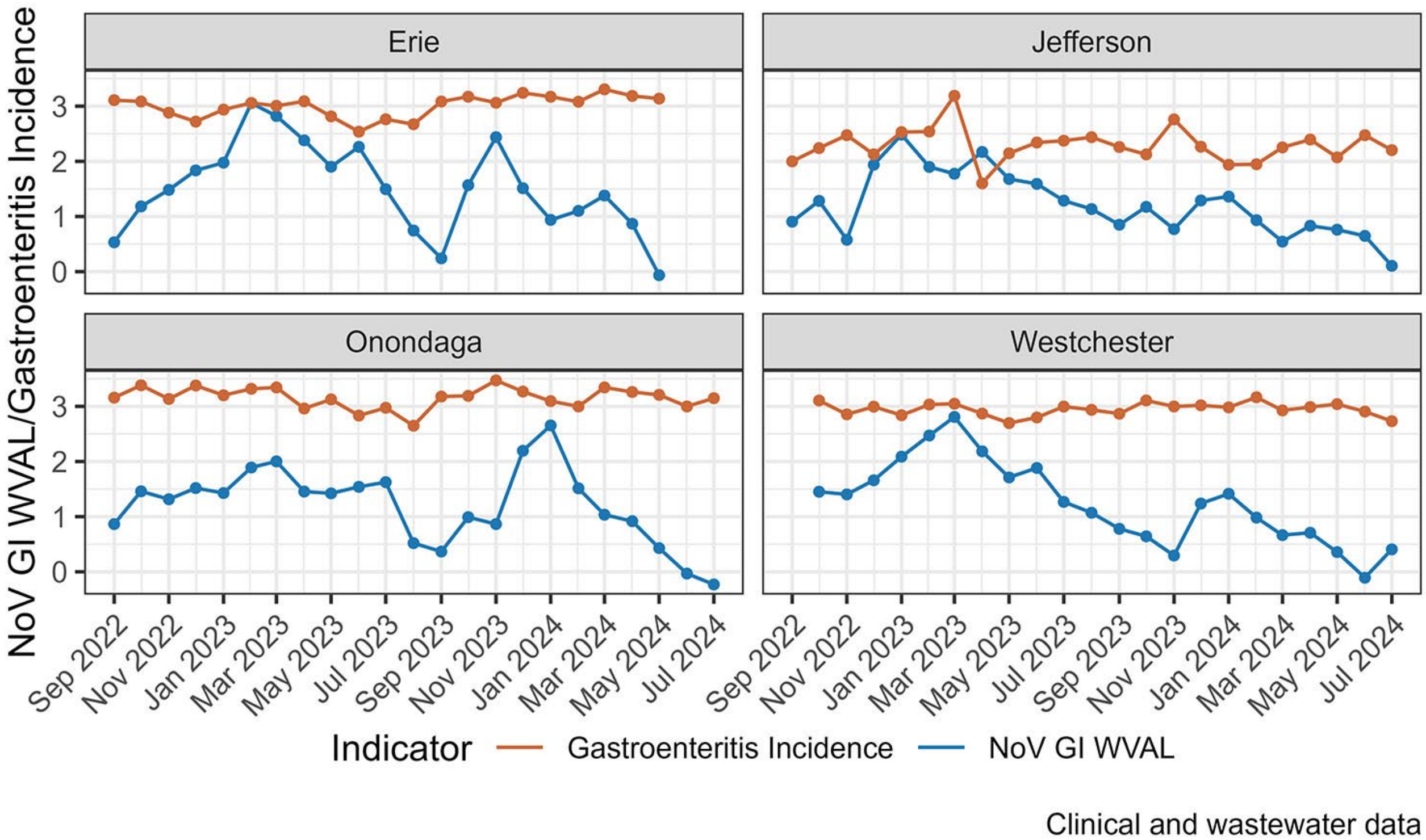

Appendix Figure 3. Temporal trends of norovirus (NoV) wastewater activity levels (WVAL) and hospitalization incidence across four New York State counties from September 2022 through July 2024. Monthly trends of population-weighted NoV GI WVAL (blue) overlaid with weekly all-cause acute gastroenteritis (AGE) hospitalization incidence per 100,000 population (orange) by county.

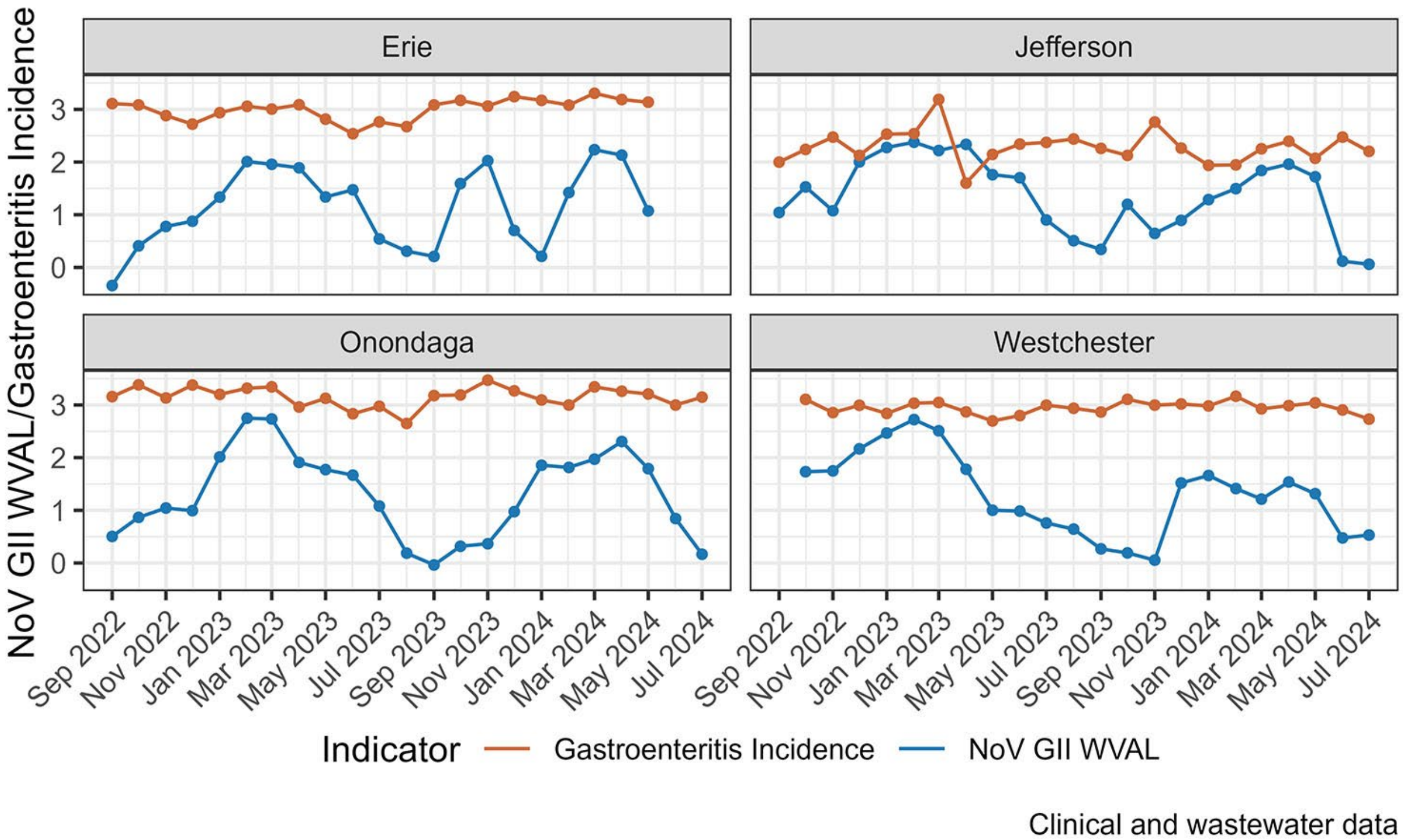

Appendix Figure 4. Temporal trends of norovirus (NoV) wastewater activity levels (WVAL) and hospitalization incidence across four New York State counties from September 2022 through July 2024. Monthly trends of population-weighted NoV GII WVAL (blue) overlaid with weekly all-cause AGE hospitalization incidence per 100,000 population (orange) by county. WVAL represents a normalized measure of NoV activity in wastewater, where higher values indicate greater NoV activity.

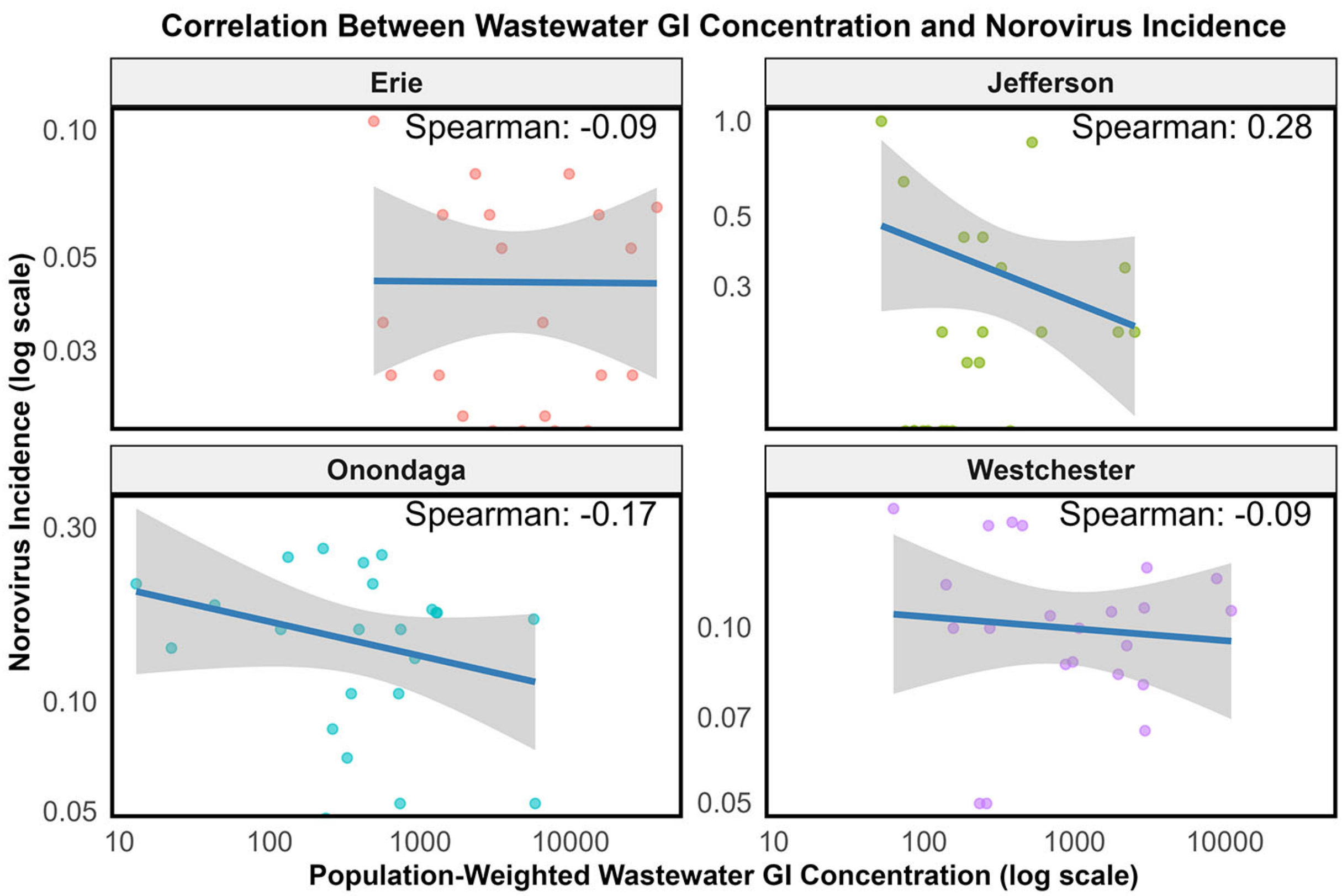

Appendix Figure 5. Spearman correlation between monthly population-weighted wastewater norovirus (NoV) concentrations and hospitalization incidence across four New York State counties. Correlation shown between monthly population-weighted wastewater NoV GI concentration and monthly NoV hospitalization incidence per 100,000 population. The blue line represents a linear regression fit with 95% confidence interval (shaded). Spearman correlation coefficients (ρ) are shown for each county within each panel.

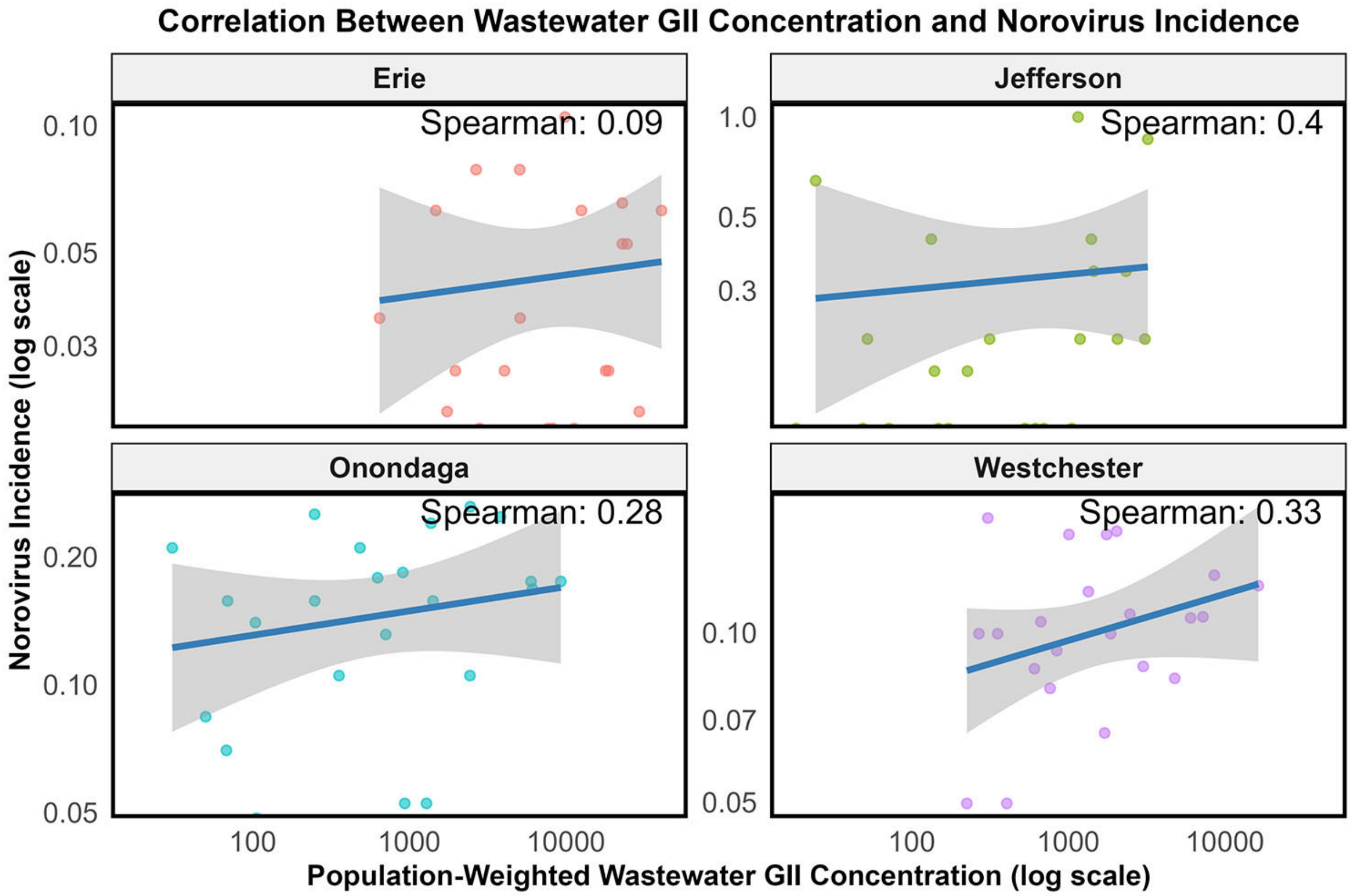

Appendix Figure 6. Spearman correlation between monthly population-weighted wastewater norovirus (NoV) concentrations and hospitalization incidence across four New York State counties. Correlation shown between monthly population-weighted wastewater NoV GII concentration and monthly NoV hospitalization incidence per 100,000 population. The blue line represents a linear regression fit with 95% confidence interval (shaded). Spearman correlation coefficients (ρ) are shown for each county within each panel.

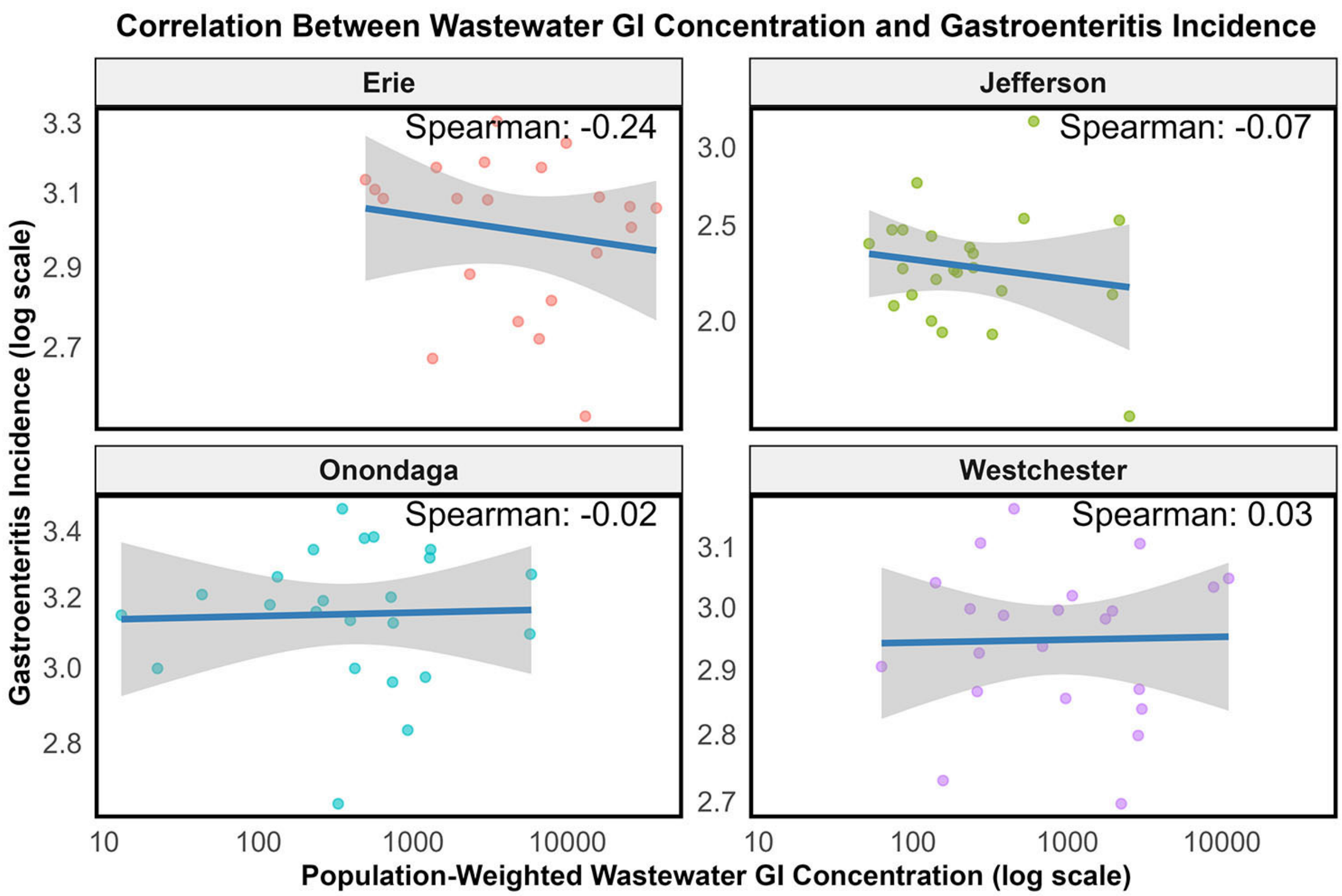

Appendix Figure 7. Spearman correlation between monthly population-weighted wastewater norovirus (NoV) concentrations and hospitalization incidence across four New York State counties. Correlation shown between monthly population-weighted wastewater GI concentration and monthly all-cause acute gastroenteritis (AGE) hospitalization incidence per 100,000 population. The blue line represents a linear regression fit with 95% confidence interval (shaded). Spearman correlation coefficients (ρ) are shown for each county within each panel.

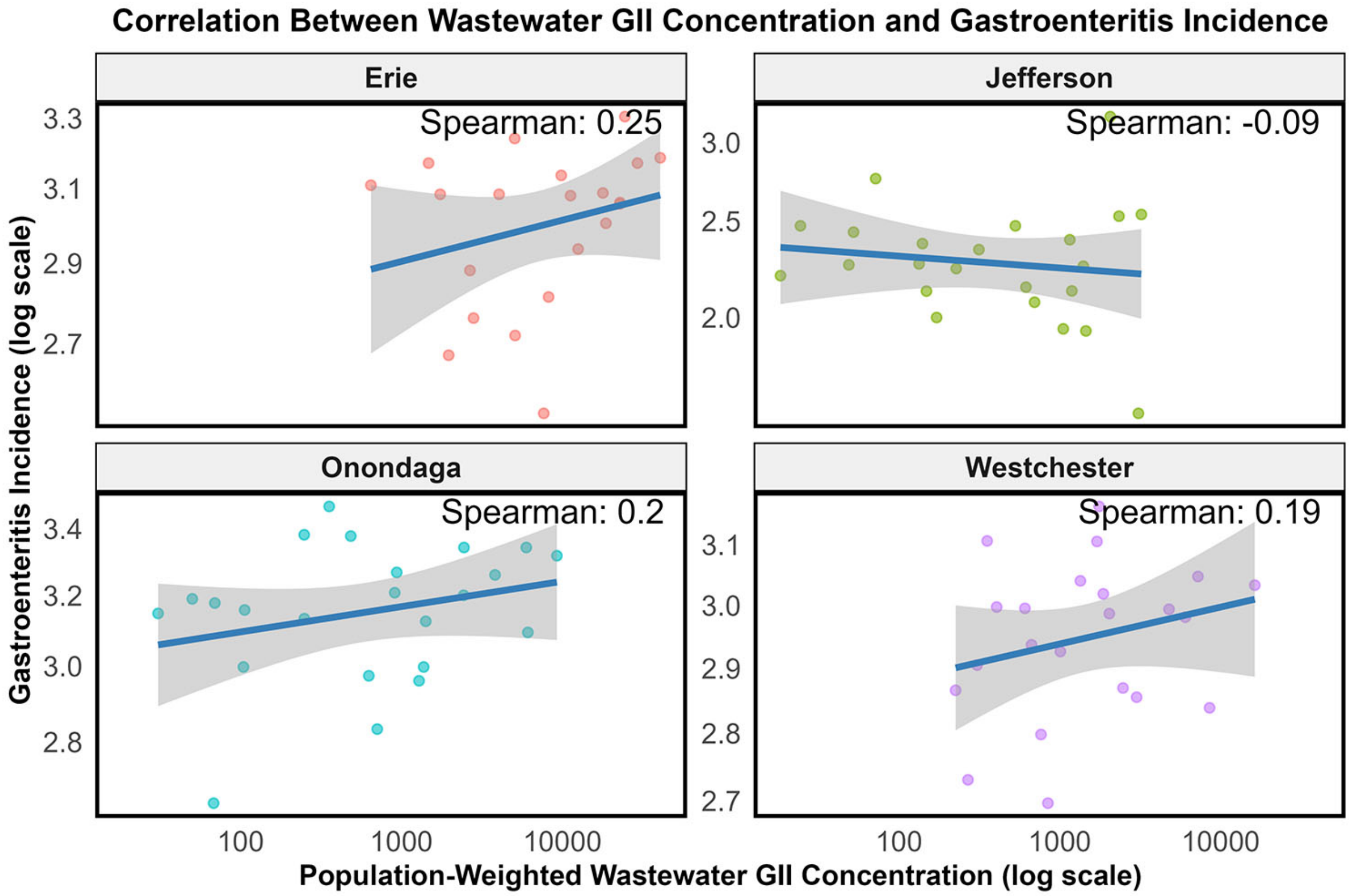

Appendix Figure 8. Spearman correlation between monthly population-weighted wastewater norovirus (NoV) concentrations and hospitalization incidence across four New York State counties. Correlation shown between monthly population-weighted wastewater GII concentration and monthly all-cause acute gastroenteritis (AGE) hospitalization incidence per 100,000 population. Both axes are on a log scale. Each point represents 1 monthly observation, colored by county. The blue line represents a linear regression fit with 95% confidence interval (shaded). Spearman correlation coefficients (ρ) are shown for each county within each panel.

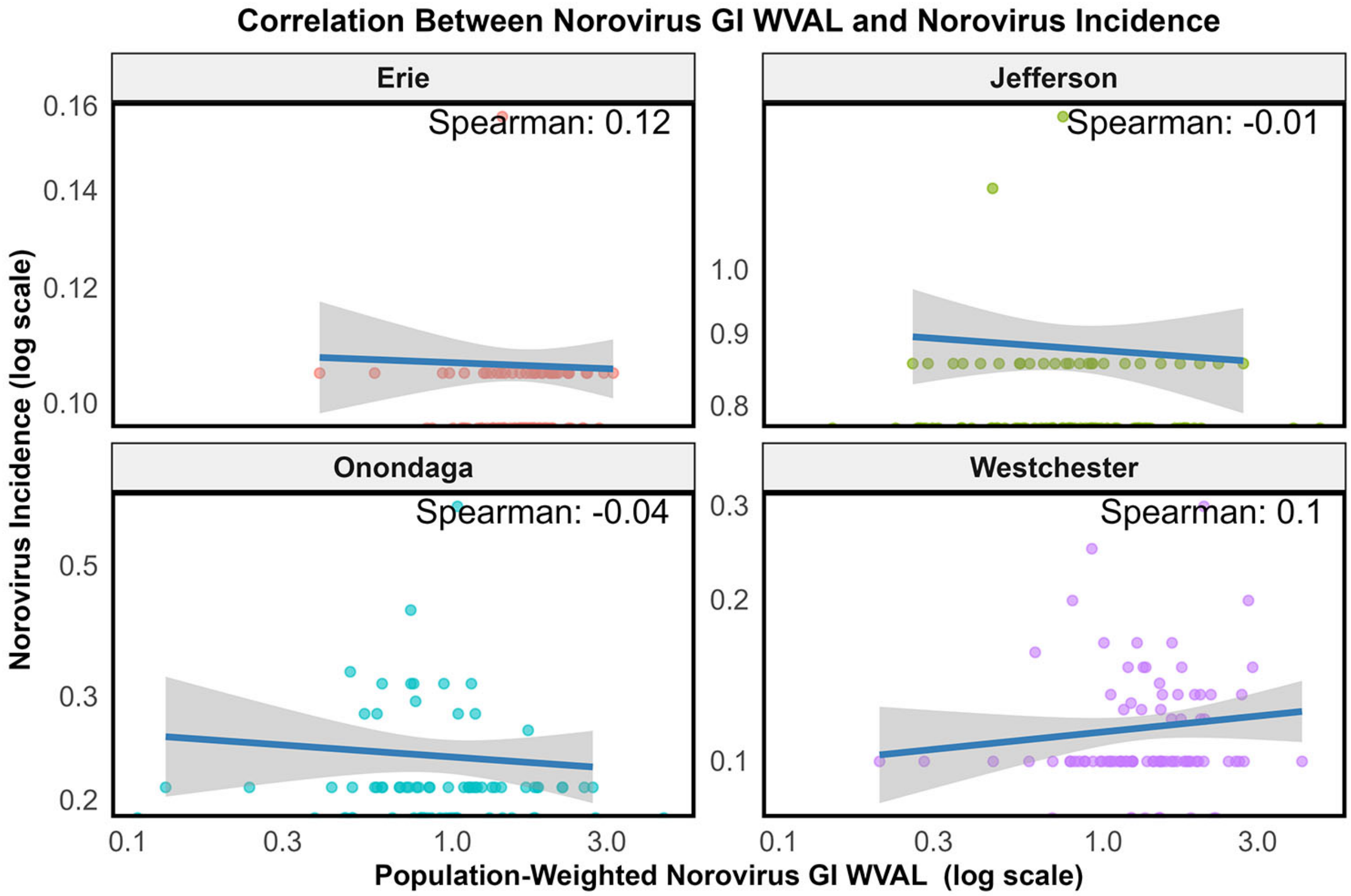

Appendix Figure 9. Spearman correlation between weekly population-weighted norovirus (NoV) wastewater activity levels (WVAL) and hospitalization incidence across four New York State counties. Correlation between weekly population-weighted NoV GI WVAL and weekly NoV hospitalization incidence per 100,000 population. The blue line represents a linear regression fit with 95% confidence interval (shaded). Spearman correlation coefficients (ρ) are shown for each county within each panel.

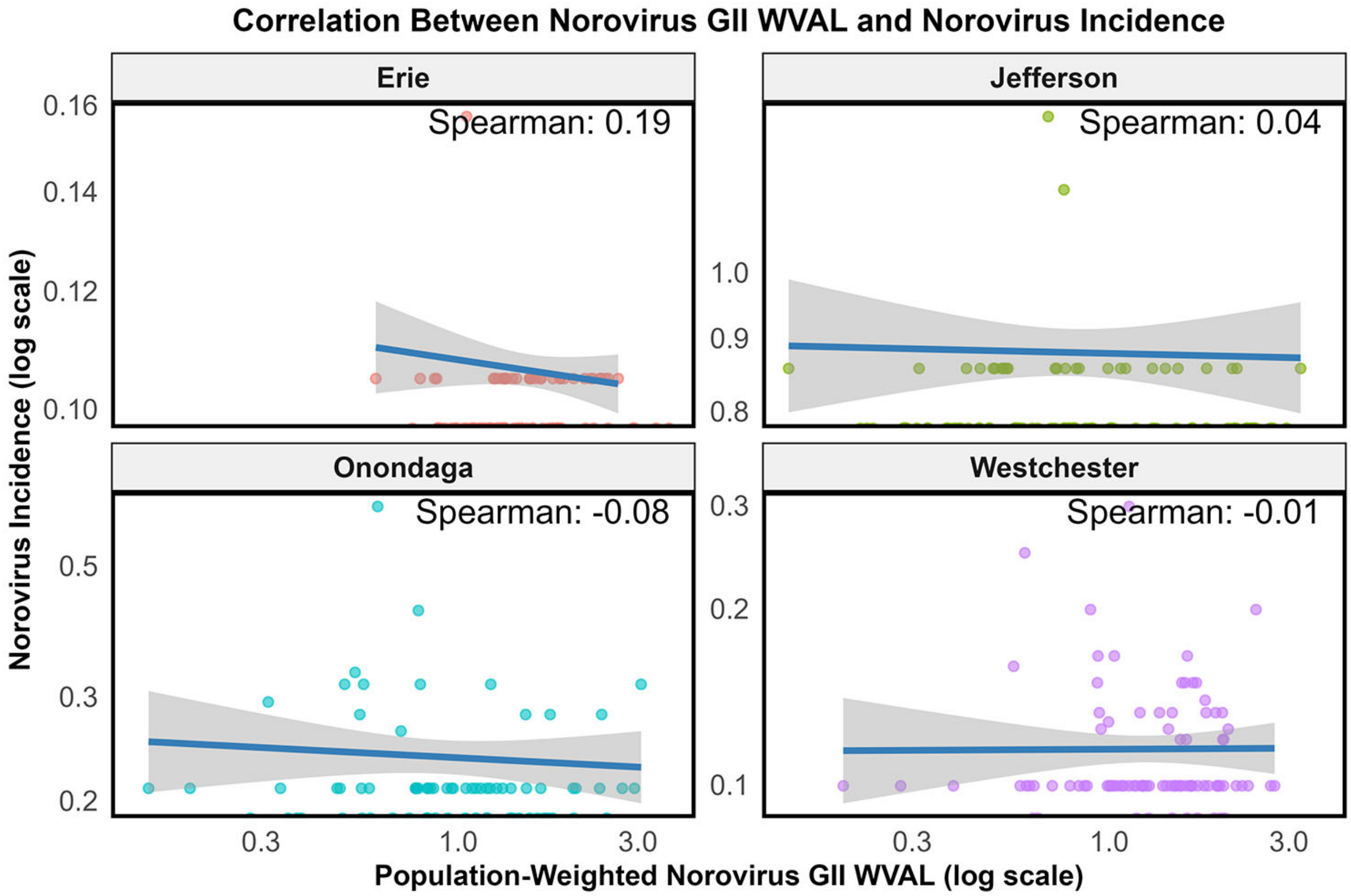

Appendix Figure 10. Spearman correlation between weekly population-weighted norovirus (NoV) wastewater activity levels (WVAL) and hospitalization incidence across four New York State counties. Correlation between weekly population-weighted NoV GII WVAL and weekly NoV hospitalization incidence per 100,000 population. The blue line represents a linear regression fit with 95% confidence interval (shaded). Spearman correlation coefficients (ρ) are shown for each county within each panel.

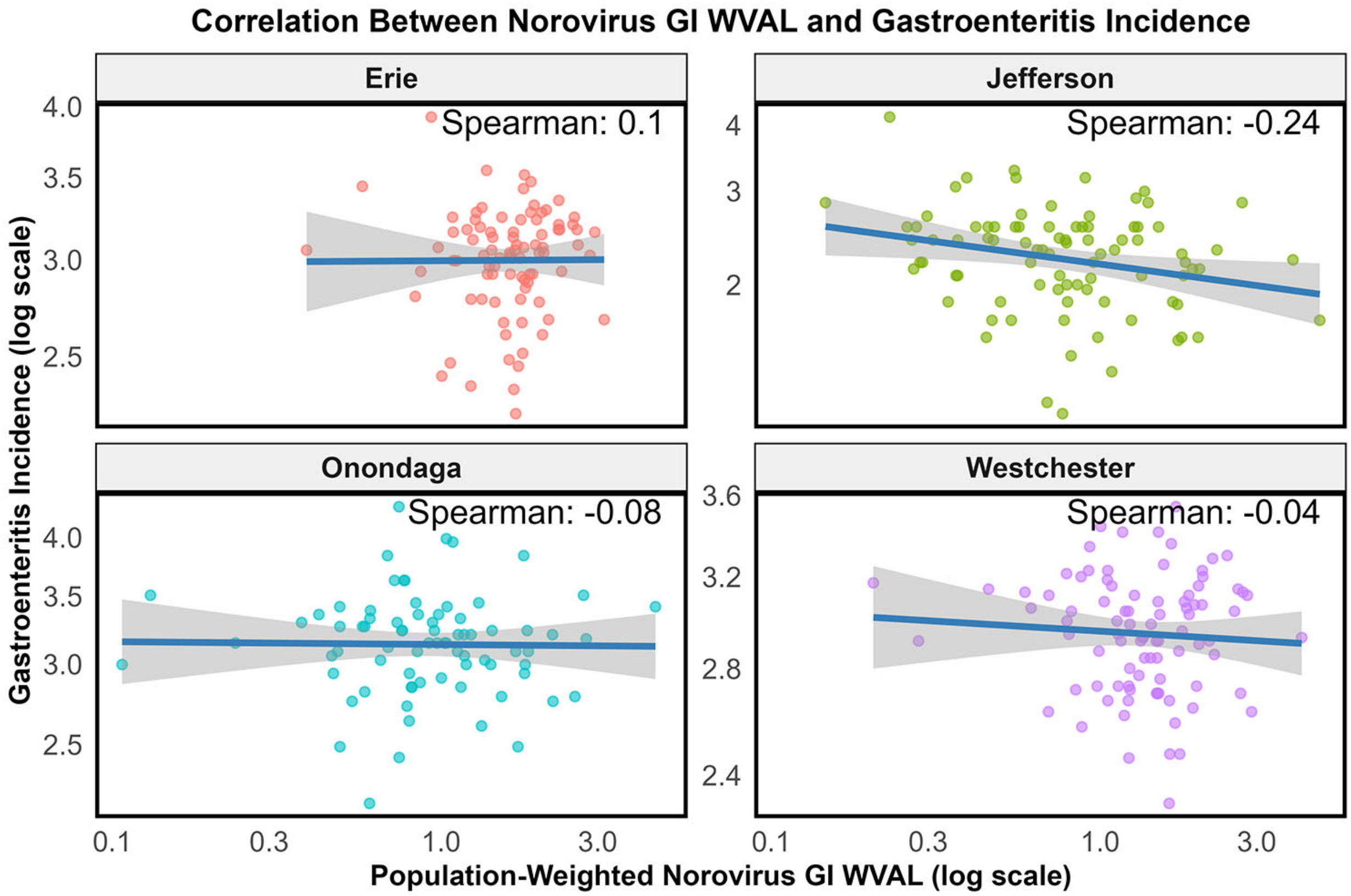

Appendix Figure 11. Spearman correlation between weekly population-weighted norovirus (NoV) wastewater activity levels (WVAL) and hospitalization incidence across four New York State counties. Correlation between weekly population-weighted NoV GI WVAL and weekly all-cause acute gastroenteritis (AGE) hospitalization incidence per 100,000 population. The blue line represents a linear regression fit with 95% confidence interval (shaded). Spearman correlation coefficients (ρ) are shown for each county within each panel.

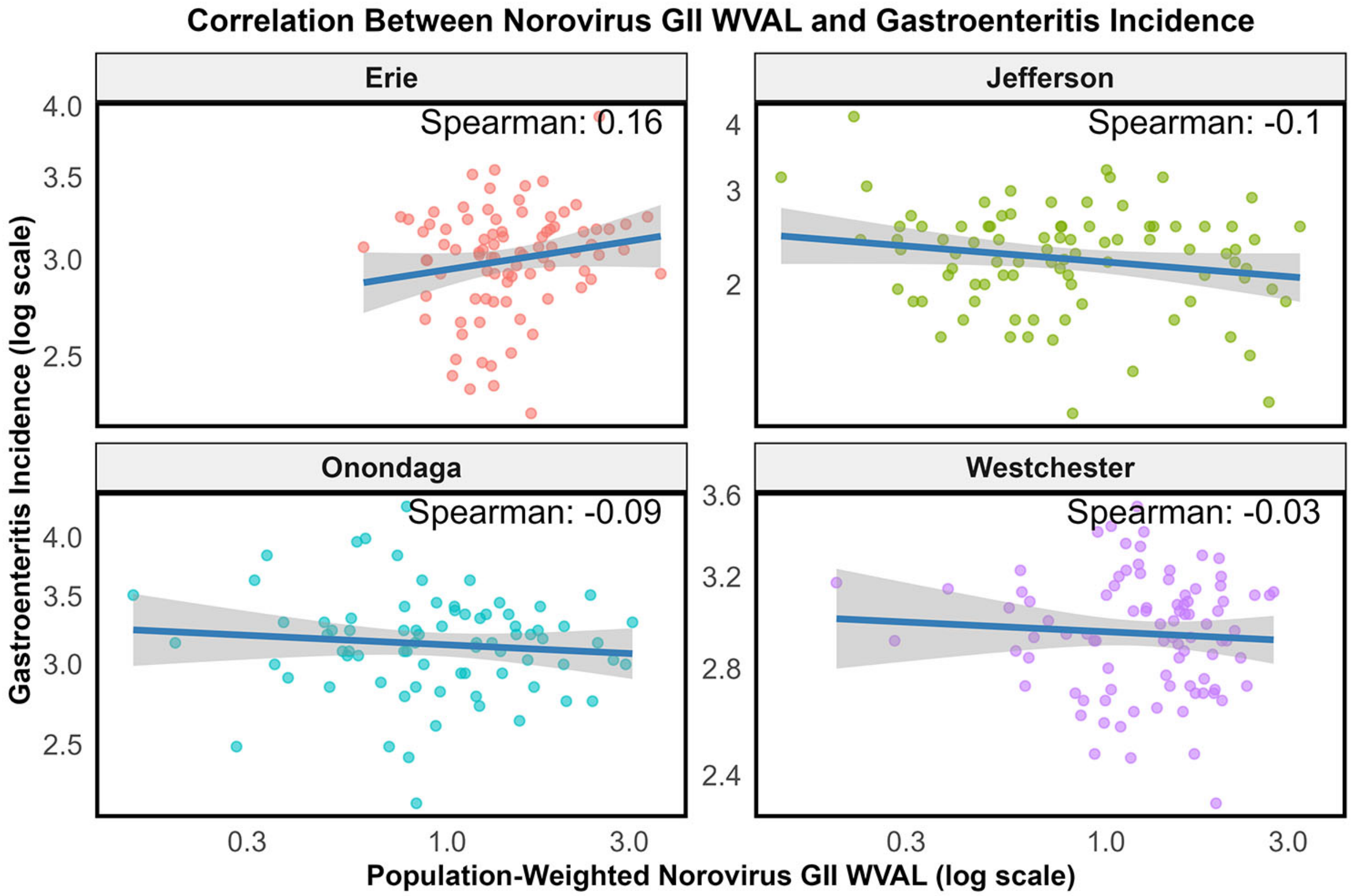

Appendix Figure 12. Spearman correlation between weekly population-weighted norovirus (NoV) wastewater activity levels (WVAL) and hospitalization incidence across four New York State counties. Correlation between weekly population-weighted NoV GII WVAL and weekly all-cause acute gastroenteritis (AGE) hospitalization incidence per 100,000 population. Both axes are on a log scale. Each point represents 1 weekly observation, colored by county. The blue line represents a linear regression fit with 95% confidence interval (shaded). Spearman correlation coefficients (ρ) are shown for each county within each panel.
